# Prediction of disease severity in young children presenting with acute febrile illness in resource-limited settings: a protocol for a prospective observational study

**DOI:** 10.1136/bmjopen-2020-045826

**Published:** 2021-01-25

**Authors:** Arjun Chandna, Endashaw M Aderie, Riris Ahmad, Eggi Arguni, Elizabeth A Ashley, Tanya Cope, Vu Quoc Dat, Nicholas P J Day, Arjen M Dondorp, Victor Illanes, Joanne De Jesus, Carolina Jimenez, Kevin Kain, Keang Suy, Constantinos Koshiaris, Estrella Lasry, Mayfong Mayxay, Dinesh Mondal, Rafael Perera, Tiengkham Pongvongsa, Sayaphet Rattanavong, Michael Rekart, Melissa Richard-Greenblatt, Mohammad Shomik, Phouthalavanh Souvannasing, Veronica Tallo, Claudia Turner, Paul Turner, Naomi Waithira, James A Watson, Mikhael Yosia, Sakib Burza, Yoel Lubell

**Affiliations:** 1 Angkor Hospital for Children, Cambodia Oxford Medical Research Unit, Siem Reap, Cambodia; 2 Centre for Tropical Medicine and Global Health, University of Oxford, Oxford, Oxfordshire, UK; 3 Médecins Sans Frontières Operational Centre Barcelona, Barcelona, Spain; 4 Centre for Tropical Medicine, Universitas Gadjah Mada, Yogyakarta, Daerah Istimewa Yogyakart, Indonesia; 5 Microbiology Department, Lao-Oxford-Mahosot Hospital-Wellcome Trust Research Unit, Vientiane, Vientiane, Lao People's Democratic Republic; 6 Faculty of Tropical Medicine, Mahidol-Oxford Tropical Medicine Research Unit, Bangkok, Thailand; 7 Hanoi Medical University, Hanoi, Viet Nam; 8 Clinical Trials, Epidemiology and Biostatistics, Research Institute for Tropical Medicine, Muntinlupa City, Philippines; 9 Laboratory Medicine & Pathobiology, University of Toronto, Toronto, Ontario, Canada; 10 Angkor Hospital for Children, Siem Reap, Siem Reap, Cambodia; 11 Department of Primary Care Health Sciences, University of Oxford, Oxford, UK; 12 Faculty of Postgraduate Studies, University of Health Sciences, Vientiane, Lao People's Democratic Republic; 13 Centre for Nutrition and Food Security (CNFS), icddr, b, Dhaka, Dhaka, Bangladesh; 14 Savannakhet Provincial Health Department, Savannakhet, Lao People's Democratic Republic; 15 University of Pennsylvania, Philadelphia, Pennsylvania, USA; 16 Salavan Provincial Hospital, Salavan, Lao People's Democratic Republic

**Keywords:** paediatrics, primary care, public health, infectious diseases

## Abstract

**Introduction:**

In rural and difficult-to-access settings, early and accurate recognition of febrile children at risk of progressing to serious illness could contribute to improved patient outcomes and better resource allocation. This study aims to develop a prognostic clinical prediction tool to assist community healthcare providers identify febrile children who might benefit from referral or admission for facility-based medical care.

**Methods and analysis:**

This prospective observational study will recruit at least 4900 paediatric inpatients and outpatients under the age of 5 years presenting with an acute febrile illness to seven hospitals in six countries across Asia. A venous blood sample and nasopharyngeal swab is collected from each participant and detailed clinical data recorded at presentation, and each day for the first 48 hours of admission for inpatients. Multianalyte assays are performed at reference laboratories to measure a panel of host biomarkers, as well as targeted aetiological investigations for common bacterial and viral pathogens. Clinical outcome is ascertained on day 2 and day 28.

Presenting syndromes, clinical outcomes and aetiology of acute febrile illness will be described and compared across sites. Following the latest guidance in prediction model building, a prognostic clinical prediction model, combining simple clinical features and measurements of host biomarkers, will be derived and geographically externally validated. The performance of the model will be evaluated in specific presenting clinical syndromes and fever aetiologies.

**Ethics and dissemination:**

The study has received approval from all relevant international, national and institutional ethics committees. Written informed consent is provided by the caretaker of all participants. Results will be shared with local and national stakeholders, and disseminated via peer-reviewed open-access journals and scientific meetings.

**Trial registration number:**

NCT04285021.

**Strengths and limitations of this study**

- Multicountry study with a minimum of 12 months continuous recruitment at each site to capture seasonal variation, maximise generalisability of findings and enable external geographical validation of the prediction model.
- Prioritisation of simple clinical parameters and biochemical biomarkers feasible for measurement with point-of-care tests, to ensure findings are practical for resource-limited settings.
- Follows the latest guidance in clinical prediction research to inform sample size, sampling frame, candidate predictor selection and derivation and validation of the clinical prediction model.
- Absence of international consensus definitions for severity of paediatric febrile illness that avoid circularity between candidate predictors and outcome categories and are practical for use in resource-limited settings; protocol-specified secondary analyses designed to address this gap.
- Translation of findings will require commercialisation, availability and uptake of low-cost point-of-care tests for any promising biochemical biomarkers identified and included in the clinical prediction model.

## Introduction

Febrile illness represents one of the most common reasons for parents to seek medical care for their children,^1–3^ and a proportion progress to severe disease with substantial risk of mortality.^4–6^ Distinguishing which febrile children require referral or admission to hospital from those who can safely be cared for in the community is difficult.^7^ Particularly in remote, rural environments and conflict settings, referral decisions involve complex mechanisms and incur costs and risks for both patient and provider. Better assessment and prioritisation of acutely unwell children would improve patient outcomes and reduce resource misallocation.^8–10^

In resource-constrained primary care contexts, the WHO’s Integrated Management of Childhood Illnesses (IMCI) and Integrated Community Case Management (iCCM) guidelines are often used to assess the need for facility-based care in febrile children presenting at the community level.^11 12^ However, results are inconsistent,^13^ adherence is poor^14^ and implementation of multiple syndrome-specific algorithms is impractical for many limited-skill health workers.^15^

Although a number of severity scores have been proposed to predict the likelihood that a febrile child might develop serious illness,^16–18^ most have been evaluated in hospitalised children and hence their potential to guide admission or referral decisions remains unclear. Furthermore, many of these scores include variables that are not feasible to collect in primary care, particularly in low resource settings.^19^ A recent systematic review concluded that the validity of existing paediatric triage tools is uncertain and that none are likely to be reliable in resource-constrained environments, with the lack of follow-up data for children not admitted highlighted as a major limitation of current research in this field.^20^ While some disease-specific tools have been developed,^21–23^ their application is limited as it is often only possible to ascertain a microbiological cause in a minority of febrile children.

A growing body of evidence indicates that common pathophysiological pathways, reflecting endothelial injury, immune activation and coagulopathy, are shared across a spectrum of microbial aetiologies.^24–27^ Microvascular dysfunction appears to occur early in the course of common childhood infections,^28^ raising the possibility that markers of these pathways might provide prognostic insight. Results from a recent study in Tanzanian outpatient clinics indicate that combining measurements of markers that reflect endothelial and immune activation with simple clinical assessments could aid triage of patients presenting from the community with acute febrile illness.^29^

This multicountry, prospective study will recruit 4900 paediatric inpatients and outpatients under the age of 5 years presenting with an acute febrile illness. The primary objective is to derive and geographically externally validate a prognostic clinical prediction model, combining measurements of host biomarkers and simple clinical features, to improve disease severity assessment of febrile children presenting from the community in resource-constrained settings across Asia.

## Methods and analysis

### Study design

**Figure 1:**
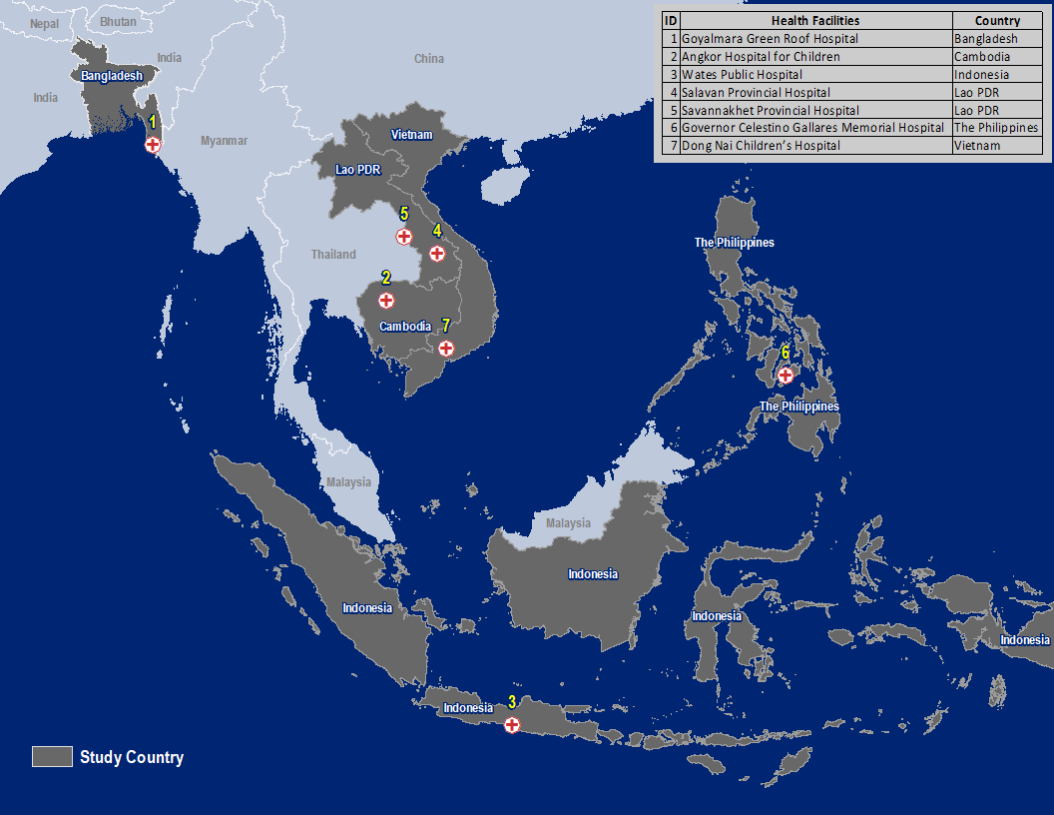
Study sites. Seven hospitals across six Asian countries where children presenting with acute febrile illness are prospectively enrolled into the study. Lao PDR = Lao People’s Democratic Republic.

This is a multicountry, observational, prospective study being conducted in Bangladesh, Cambodia, Indonesia, Laos, the Philippines and Vietnam (figure 1). The study started enrolment in March 2020 and will recruit a cohort of at least 4900 children aged between 28 days and 5 years presenting to hospital with acute febrile illness. Recruitment is stratified by the treating clinician’s decision to admit or send home: 3400 children whom the treating clinician decides to admit and 1500 children sent home directly from the outpatient department.

### Study settings

This study aims to develop a prognostic clinical prediction tool to improve assessment and prioritisation of febrile children in rural, hard-to-reach settings and decentralised models of care across Asia. However, derivation of a prediction model requires a certain number of ‘outcome events’ (participants who progress to develop serious illness), and hence recruiting children presenting at the most peripheral levels of the health system would be challenging. To overcome this, midlevel hospitals (equivalent to the district or provincial level) were selected as study sites, acknowledging that differences in care-seeking patterns between community health facilities and hospitals exist.

This compromise risks a potential loss of generalisability to community settings, the ultimate intended-use setting for the prediction tool. To mitigate this risk, study sites were identified which serve as primary points of access for a predominantly rural and underserved population, the demographics of which are representative of patients presenting to lower levels of care. Hence, as far as possible, we hope to have ensured that the primary difference between the study sites and eventual intended-use sites is the frequency with which children at risk of serious illness attend, rather than systematic differences in their demographic characteristics. This will maximise the chance of successful out-of-sample validation and generalisability of the tool to community settings.

### Eligibility criteria

Children within the target age range are eligible to participate if they meet all of the following inclusion criteria: (1) Their caregiver is willing and able to provide informed consent for their participation; (2) They have an axillary temperature ≥37.5°C OR <35.5°C or history of fever in the last 24 hours and (3) The onset of their illness occurred ≤2 weeks ago.

Children are not eligible to participate if they meet any of the following exclusion criteria: (1) An accident or trauma is the reason for their presentation; (2) They are presenting ≤3 days after routine immunisations; (3) They have known specific comorbidities (including, immunosuppression, active chronic infection or major cardiorespiratory conditions); (4) They have been admitted overnight at any health facility during the current illness or (5) They have received >15 min of inpatient treatment (intravenous or nebulised medications or supplemental oxygen) at the study site prior to being screened for study eligibility. To maximise diversity within the study population, participants can only be enrolled once.

### Participant enrolment

At enrolment (day 0), demographic data, perinatal and historical information and presenting clinical symptoms are collected via interview with the participant’s caretaker. Anthropometric data, vital and clinical signs are measured by the research team (see below). All data are captured on electronic case record forms (eCRFs) using mobile Android tablets via Open Data Kit Collect software. A summary of study enrolment and assessment procedures is provided in the online supplemental file (S1).

10.1136/bmjopen-2020-045826.supp1 Supplementary data

A venous blood sample is collected for batched retrospective off-site measurement of a panel of prespecified host biomarkers (table 1) and targeted aetiological investigations (table 2). In addition, a nasopharyngeal swab is collected for detection of common viral pathogens. Participants are provided with routine care, as determined by the treating clinician. Blood cultures are collected when clinically indicated, processed on site (or at a nearby quality-assured laboratory) and results fed back to the treating clinical team. Where necessary, diagnostic stewardship training is provided to encourage clinically appropriate utilisation of blood cultures and assist with interpretation of antimicrobial susceptibility testing results.

**Table 1:** Candidate host biomarkers

Host biomarker	Summary of supportive data
Angiopoietin-1 and 2 (Ang-1 and Ang-2)	Supportive data from Asia/SSA in children/adults, that Ang-2, Ang-1 and/or Ang-2:1 ratio predicts mortality in malaria, SBI and all-cause febrile illness.^26 29 46–49^
Soluble fms-like tyrosine kinase-1 and 2 (sFlt-1 and s-Flt-2)	Supportive data from SSA that sFlt-1 predicts mortality in paediatric severe malaria and adults with all-cause febrile illness^26 29 46^; in Thailand sFlt-2 discriminates uncomplicated dengue from dengue associated with plasma leak in children.^50^
Soluble vascular adhesion molecule-1 (sVCAM-1)	Supportive data from SSA that sVCAM-1 predicts mortality in children/adults with all-cause febrile illness.^26 29^
Soluble intercellular adhesion molecule-1 (sICAM-1)	Supportive data from Uganda that sICAM-1 predicts mortality in paediatric severe malaria and all-cause febrile illness^26 51^; in Bangladesh, sICAM-1 predicts mortality in neonatal sepsis.^52^
Soluble tumour necrosis factor receptor-1 (sTNFR-1)	Supportive data from Tanzania that sTNFR-1 predicts mortality in children/adults with all-cause febrile illness.^26 29^
Soluble thrombomodulin (sTM)	Supportive data from Malawi that sTM predicts mortality in children with severe malaria.^53^
C-X-C motif chemokine-10 (CXCL-10)/interferon-y induced protein-10 (IP-10)	Supportive data from Uganda that IP-10 predicts mortality in children with severe malaria.^46^
Soluble triggering receptor expressed on myeloid cells-1 (sTREM-1)	Supportive data from SSA that sTREM-1 predicts mortality in paediatric severe malaria and in adults/children with all-cause febrile illness^26 29 46 51^; in Asia, sTREM-1 predicted length of stay in infant febrile illness and in-hospital mortality in adults hospitalised with infection.^54 55^
Interleukin-6 (IL-6)	Supportive data from India that IL-6 is predictive of mortality in children with dengue^56^; in Switzerland, supportive data that IL-6 predicts duration of antibiotic therapy for febrile children with lower respiratory tract infections.^57^
Interleukin-8 (IL-8)	Supportive data from India that IL-8 is predictive of mortality in children with dengue^56^; in the UK, supportive data that IL-8 predicts disease severity in children with meningococcal disease.^58^
Interleukin-10 (IL-10)	Supportive data from India that IL-10 is predictive of mortality in children with dengue.^56^
Chitinase-3-like protein-1 (CHI3L1)	Supportive evidence from SSA that CHI3L1 is predictive of mortality in children/adults with all-cause febrile illness.^26 29^
Procalcitonin (PCT)	Supportive evidence that PCT is predictive of severe illness in hospitalised children with suspected bacterial infections or meningococcal disease.^59 60^
Lactate	Supportive evidence that lactate is predictive of mortality in hospitalised children with febrile illness in East Africa.^61 62^
Glucose	Supportive evidence that hypoglycaemia is predictive of mortality in hospitalised children in Tanzania.^63^
Haemoglobin	Supportive evidence that haemoglobin is predictive of mortality in hospitalised children with febrile illnesses in East Africa.^62 64^
C reactive protein (CRP)	Although there is limited supportive evidence for the use of CRP as a prognostic marker for disease severity, as it is the most widely studied biomarker in our region, and numerous point-of-care tests already exist, further evaluation is warranted.

List is subject to review as new evidence comes to light during the conduct of the study. SBI = serious bacterial infection; SSA = sub-Saharan Africa.

**Table 2:** Planned aetiological investigations

Pathogen	Platform	Specimen type
Dengue virus	PCR	Venous blood
Chikungunya virus	PCR	Venous blood
Pan-Flavivirus	PCR	Venous blood
Pan-Alphavirus	PCR	Venous blood
*Orientia tsutsugamushi*	PCR	Venous blood
*Rickettsia* spp	PCR	Venous blood
*Leptospira* spp	PCR	Venous blood
Eubacteria (16 s rDNA)	PCR	Venous blood
Influenza A virus	PCR*	Nasopharyngeal swab
Influenza B virus	PCR*	Nasopharyngeal swab
Respiratory syncytial virus	PCR*	Nasopharyngeal swab
Bacterial bloodstream infection	Blood culture	Venous blood

Blood cultures are collected at the discretion of the treating clinician and results provided to the treating clinical team. All other aetiological investigations are performed retrospectively using standardised protocols at reference laboratories.

*Nasopharyngeal swab specimens will be tested using the BioFire FilmArray Respiratory Pathogen 2 panel which includes a broader range of aetiological targets (www.biofiredx.com/products/the-filmarray-panels/filmarrayrp).^65^ However, as causality can be difficult to determine for some of these agents, they have not all been named here.

Equipment for measurement of clinical parameters (pulse oximeters (Masimo Rad-5V), respiratory rate counters, weighing scales (seca 874), height/length boards, axillary thermometers and mid-upper arm circumference tapes) were procured centrally and distributed to the study sites to ensure standardisation. Data from the eCRFs are uploaded at the end of each day to a secure server located at the Mahidol-Oxford Tropical Medicine Research Unit (MORU) in Bangkok, Thailand. Prior to commencing recruitment at each site, site initiation visits including training in the study’s standard operating procedures (SOPs) and ensuring the study is conducted in accordance with Good Clinical Practice, are conducted by MORU’s Clinical Trials Support Group. Monitoring is conducted at specified intervals to ensure compliance with the study protocol and perform source data verification checks.

### Sample management and laboratory assessments

Participants’ nasopharyngeal swabs and venous blood samples (collected in ethylenediaminetetraacetic acid and fluoride oxalate tubes) are transported on ice to the onsite laboratory. Samples are processed within a maximum of 4 hours and the nasopharyngeal specimens and blood aliquots (whole blood and plasma) are stored at −20°C for a maximum of 1 month before being transported on dry ice to an in-country −80°C freezer within the vicinity of the study site. Samples are shipped on dry ice at 6 monthly intervals to MORU’s central reference laboratories in Bangkok, Thailand.

Multianalyte assays will be used for quantification of host biomarkers (table 1) in plasma as previously described.^30^ Biomarker selection has been informed by systematic review of the available evidence,^31 32^ ensuring that assays with the highest likelihood of translation into clinical practice in settings similar to which the study is being conducted are prioritised. Molecular diagnostics (multiplexed PCR) will be performed on whole blood and nasopharyngeal specimens to identify common bacterial and viral causes of febrile illness (table 2).

### Recruitment strategy and sample frame

Participants are recruited from the outpatient and emergency departments of the study sites. Recruitment is planned over a minimum continuous 12-month period at each site to ensure seasonality is adequately captured. Recruitment reports are generated by the MORU data management team, disseminated to the research team and discussed at monthly data review meetings attended by the study management group (including the site principal investigators, central coordinating team and study statisticians).

During the hours of study recruitment, all non-elective admissions of children aged between 28 days and 5 years are screened for eligibility. Caregivers of eligible children are asked to provide informed consent and participant enrolment is consecutive. The recruitment rate is monitored by the study management group.

Children sent home directly from the hospital outpatient department are selected randomly (using lists generated by the study statisticians) and screened for eligibility. The recruitment rate is monitored and adjusted to ensure that the recruitment period of children sent home directly from the outpatient department mirrors that for admitted children at each site.

### Screening weeks

During 3 weeks each year, the research team screen and determine eligibility of all (or if infeasible, a representative sample of) children aged between 28 days and 5 years presenting to the study site during the hours of recruitment. Screening weeks are spaced throughout the year to ensure seasonal variation in patient attendance is captured. These data will be combined with the daily routinely collected hospital data to estimate the total number of eligible children presenting to the study site. This information will be used to weight the regression analysis to derive the prediction model (see the Statistical analysis section).

### Participant follow-up and outcome measurement

All children are followed up by the research team on day 2 (window period:+2 days) and day 28 (window period +7 days). Follow-up is conducted face to face (via return to the study site or community outreach visit) or via telephone, depending on the constraints at the different study sites. The clinical outcome of the acute febrile illness is recorded, including the details of any further care sought between enrolment and the follow-up contact. In the event that a participant is uncontactable, a minimum of two further contacts are attempted during the window period before a participant is declared lost to follow-up. In addition, admitted children are followed up each day for the first 2 days of their admission and on the day of discharge. Information on the treatment administered by the clinical team, as well as discharge diagnosis, are extracted from the participant’s medical record. A full schedule of enrolment and assessments is provided in the online supplemental file (S1).

Outcome categories are ordinal (1–4) and calculated on day 28 (window period:+7 days) using a composite of vital status, receipt or referral for organ support (defined as mechanical or non-invasive ventilation, receipt of inotropic therapy or renal replacement therapy), length of inpatient hospital stay (at the study site or other health facility) and persistence of symptoms present at enrolment (table 3).

**Table 3:** Outcome categories

Outcome Category	Definition
1	Death or receipt of organ support* ≤48 hours after enrolment
2	Death >48 hours after enrolment and before D28 AND did not meet criteria for severe disease *or* Admitted for >48 hours at any health facility before D28 AND did not meet criteria for severe disease
3	Admitted for ≤48 hours at any health facility before D28 AND did not meet criteria for severe or probable severe disease *or* Not admitted to any health facility AND ongoing symptoms at D28
4	Not admitted to any health facility AND symptoms resolve by D28

*Organ support defined as receipt of or referral for mechanical or non-invasive ventilation, inotropic support or renal replacement therapy.

### Sample size considerations

By using a conservative estimate of R^2^, a shrinkage factor of 0.9 and a prevalence of severe outcomes (outcome category 1) of 13%,^33 34^ we estimated that we would need approximately 14 events per parameter for derivation of the prediction model.^35^ The derivation dataset will consist of at least 3600 children, with oversampling of those more likely to develop a severe outcome (2400 admitted children and 1200 children whom the treating clinician decides to send home without admission). Based on our estimated prevalence, we would expect to recruit 280 children who progress to meet the primary endpoint (outcome category 1), permitting evaluation of up to 20 candidate predictors, while minimising the risk of overfitting and allowing for up to a 10% attrition rate. This sample size is the minimum number of children that we aim to recruit. If feasible, we will allow for the possibility of recruiting a higher number of participants, as this will permit inclusion of more candidate predictors in the model.

The validation dataset will be geographically distinct and will consist of at least 1000 admitted children and 300 children sent home without admission, providing a total (enriched) sample of 1300 children. Based on the same estimated prevalence and attrition rate, this would provide us with at least the required 100 outcome events in the validation dataset.^36^

### Statistical analysis

Descriptive analysis of presenting syndromes, fever aetiologies, clinical outcomes as well as candidate predictors (baseline clinical and biochemical parameters) will be carried out. Reasons for important discrepancies will be explored across sites and the derivation dataset for the prediction model will be defined. This derivation dataset will contain the outcomes (table 3), baseline clinical data and host biomarkers (table 1) to be included in the prediction model. Preliminary covariate selection has already been informed by subject knowledge using systematic literature review and expert judgement.^31 32 37^

Penalised ordinal logistic regression (or its multinomial equivalent if assumptions about proportionality between outcome categories are not met) will be used for further covariate selection in order to determine the final model. For continuous variables, transformations will be used if necessary. If feasible, bootstrapping will be used to estimate model performance (discrimination and calibration) and estimate the amount of optimism in the derivation dataset.

The final model obtained from the derivation dataset will be applied on the validation dataset and its performance will be evaluated. The c-statistic will be used to examine discrimination and calibration plots for calibration. The performance of the model at different predicted probability thresholds will be examined.

A full case analysis will be conducted if the overall amount of missing data is less than 5%. If the fraction of missing data is more than 5% then multiple imputation will be used and regression estimates will be combined using Rubin’s rule. Imputation will be conducted separately for the derivation and validation datasets.

The performance of the prediction model derived in the primary analysis will be examined in children presenting with specific clinical syndromes (eg, acute respiratory infections, diarrhoeal disease or acute undifferentiated fever) and fever aetiologies. Performance will be reported using classification tables (confusion matrices) of observed probabilities against predicted probabilities.

Additional protocol-specified secondary analyses will be conducted using alternative approaches for outcome classification (eg, binary and continuous) to explore the impact on the development of the prediction model (see the Discussion section).

### Patient and public involvement

Prior to finalisation of the study protocol, the concept for the research, study design and sample collection procedures were presented to the Young Persons’ Advisory Group at the Angkor Hospital for Children, Siem Reap, Cambodia. This group, comprising around twenty children aged 10–15 years, provided feedback on the project to ensure alignment with the priorities of the population the research is intended to benefit.

## Discussion

This prospective study will recruit at least 4900 children across seven hospitals in six Asian countries; measure a broad panel of clinical and biochemical biomarkers; and follow participants up over an extended period to determine clinical outcome. It will then follow the latest guidance in clinical prediction model building to derive and geographically externally validate a prognostic clinical prediction model to assist community healthcare providers assess the need for facility-based medical care in children presenting with acute febrile illness in resource-constrained settings across Asia.

Despite increasing interest in clinical prediction research, many studies have limited impact.^38^ This study was designed following the latest guidance^39^: the sample size calculation and recruitment strategy (sampling frame) are based on recent methodological advances,^40 41^ and selection of candidate predictors was informed by expert consensus, feasibility and systematic review of the existing evidence.^31 32 37 42^ Nonetheless, several aspects warrant discussion.

The eligibility criteria for this study are based around fever, yet many children, particularly younger infants, may not mount a fever in response to serious infection. By broadening the eligibility criteria to include hypothermia and history of fever we believe this risk will be partly mitigated. We expect to capture the majority of children with acute infectious illness and hope that ‘abnormal temperature or history of fever’ (rather than, for example, ‘clinician-suspected infection’) will provide a ‘pragmatic point-of-entry’, feasible for lesser-trained community health workers, for use of the clinical prediction tool in the future.

The study will only recruit children aged between 28 days and 5 years, limiting our ability to develop a parsimonious model for all children presenting with suspected infection. In particular, neonates, who carry a disproportionate risk of sepsis, are excluded.^43^ This decision reflects the fact that all febrile neonates require further assessment, and that outside the neonatal period the greatest burden occurs in children under the age of 5 years. Including children of all ages would have required substantially greater resources to ensure adequate power to examine the interaction of predictive performance with age.

Developing a prediction model in settings in which the outcome of interest (in this case, episodes of severe febrile illness) occurs at relatively low frequency poses unique challenges, in particular how best to obtain sufficient precision without requiring an unfeasibly large sample size.^41^ Our stratified recruitment strategy ‘oversamples’ admitted children and provides an ‘enriched’ sample with more ‘outcome events’. This permits evaluation of a greater number of candidate predictors, without increasing the risk of overfitting the prediction model.^40^ Triangulating study data with data from the screening weeks and routine hospital records will provide the necessary information to estimate the denominator (total number of eligible children presenting to the study sites) and weight the regression analysis to develop a prediction model applicable to lower-prevalence community settings.

A further challenge is the choice of outcome (reference standard). We purposively opted not to focus solely on predicting mortality. Although mortality is a ‘hard’ outcome, predicting death may be of limited utility, compared with predicting severe, and in many instances treatable, illness. Furthermore, mortality occurs infrequently and is influenced (mediated) by the level and quality of care, for example, the experience of the healthcare workers and access to interventions such as oxygen, fluids, antibiotics, etc. Estimating a generalisable prediction model (generalisable outside of the studied settings) would necessitate adjustment for the correct set of mediating variables. Adjustment for mediating variables is difficult and can introduce selection bias. To avoid this issue, we have designed ordinal outcome categories, which group children according to the eventual severity of their illness, assessed throughout until day 28.

We recognise that these categories are imperfect, for example, children may remain in hospital for longer than 48 hours for reasons other than illness severity, and outcome misclassification will underestimate the predictive performance of candidate predictors (index tests).^44^ Our protocol-specified analyses are intended to explore this further. We will look to derive a prediction model using data from the subset of children with severe (outcome category 1) and non-severe (outcome category 4) illnesses only (table 3). These binary outcome categories will be less sensitive to misclassification but may not adequately discriminate among more moderately unwell children.^45^ We will, therefore, also develop and validate a pre-specified scale that quantifies illness severity on a continuum.

This study is a first step to developing a tool that a community healthcare provider could use to guide their assessment of whether a febrile child requires referral or admission for facility-based medical care. Operationalising the prediction model developed during this study will require adaptation of the algorithm to electronic and/or paper-based decision-support tools, development of low-cost point-of-care tests for any promising biomarkers for which tests do not already exist, and iterative design in partnership with community health workers and ministries of health. Implementation will need to be supported by development of health worker capacity and contextualised to the insecure contexts in which a tool like this is most urgently needed.

## Ethics and dissemination

This study (protocol V.2.0; 21 January 2020) has received ethical approval from the Oxford Tropical Research Ethics Committee (OxTREC reference: 59–19), the Médecins Sans Frontières Institutional Review Board (MSF IRB reference: 1967) and the relevant institutional and national ethics committees of each participating country. Any necessary protocol amendments will be approved by these same ethics committees prior to implementation. Written, informed consent to participate (and for the storage of clinical data and biological specimens for use in future ethically approved studies with similar aims) is obtained from the parent or legally acceptable representative of all participants.

The study protocol, informed consent materials, SOPs, data management plan and the datasets generated and/or analysed during the current study, are freely available from the MORU and MSF Data Access Committees on reasonable request. Results will be shared with local and national stakeholders, including the local communities at each of the study sites, and disseminated via peer-reviewed open-access journals and scientific meetings.

## Supplementary Material

Reviewer comments

Author's manuscript
