# Supplementary material for: Prediction of disease severity in young children presenting with acute febrile illness in resource-limited settings: a protocol for a prospective observational study
Source: BMJ Open. 2021 Jan 25;11(1):e045826. doi: 10.1136/bmjopen-2020-045826 (PMC7839891; doi:10.1136/bmjopen-2020-045826)
Supplement: Supplementary data [file bmjopen-2020-045826supp001.pdf]

S1. Schedule of enrolment and assessments.

	STUDY PERIOD				
	Enrolment	Follow-up			
TIMEPOINT	DAY 0	DAY 1	DAY 2	DISCHARGE	DAY 28
**ENROLMENT**					
Eligibility screen	X				
Informed consent	X				
**ASSESSMENTS**					
Medical history	X				
Demographics	X				
Anthropometrics	X				
Clinical symptoms	X				
Clinical signs	X				
Venepuncture	X				
Nasopharyngeal swab	X				
Clinical diagnosis	•			¤	
Treatment received		¤	X	¤	X
Symptom resolution			X		X
Vital status			X	¤	X

X = all children; ¤ = admitted children only; • = children sent home directly from hospital outpatient department only
