# Supplementary material for: Prediction of disease severity in young children presenting with acute febrile illness in resource-limited settings: a protocol for a prospective observational study
Source: BMJ Open. 2021 Jan 25;11(1):e045826. doi: 10.1136/bmjopen-2020-045826 (PMC7839891; doi:10.1136/bmjopen-2020-045826)
Supplement: Author's manuscript [file bmjopen-2020-045826.draft_revisions.pdf]

BMJ Open is committed to open peer review. As part of this commitment we make the peer review history of every article we publish publicly available.

When an article is published we post the peer reviewers' comments and the authors' responses online. We also post the versions of the paper that were used during peer review. These are the versions that the peer review comments apply to.

The versions of the paper that follow are the versions that were submitted during the peer review process. They are not the versions of record or the final published versions. They should not be cited or distributed as the published version of this manuscript.

BMJ Open is an open access journal and the full, final, typeset and author-corrected version of record of the manuscript is available on our site with no access controls, subscription charges or pay-per-view fees (http://bmjopen.bmj.com).

If you have any questions on BMJ Open's open peer review process please email info.bmjopen@bmj.com

BMJ Open

Prediction of disease severity in young children presenting with acute febrile illness in resource-limited settings: a protocol for a prospective observational study

Journal:	*BMJ Open*
Manuscript ID	bmjopen-2020-045826
Article Type:	Protocol
Date Submitted by the Author:	13-Oct-2020
Complete List of Authors:	Chandna, Arjun; Cambodia Oxford Medical Research Unit, Angkor Hospital for Children; University of Oxford, Centre for Tropical Medicine and Global Health<br>Aderie, Endashaw; Médecins Sans Frontières Operational Centre Barcelona<br>Ahmad, Riris; Universitas Gadjah Mada, Centre for Tropical Medicine<br>Arguni, Eggi; Universitas Gadjah Mada, Centre for Tropical Medicine<br>Ashley, Elizabeth ; University of Oxford, Centre for Tropical Medicine and Global Health; Lao-Oxford-Mahosot Hospital-Wellcome Trust Research Unit, Microbiology Department<br>Cope, Tanya; Mahidol-Oxford Tropical Medicine Research Unit, Faculty of Tropical Medicine<br>Dat, Vu Quoc; Hanoi Medical University<br>Day, Nicholas; Mahidol Oxford Tropical Medicine Research Unit, Faculty of Tropical Medicine; University of Oxford, Centre for Tropical Medicine and Global Health<br>Dondorp, Arjen; Mahidol-Oxford Tropical Medicine Research Unit, Faculty of Tropical Medicine; University of Oxford, Centre for Tropical Medicine and Global Health<br>Illanes, Victor; Médecins Sans Frontières Operational Centre Barcelona<br>De Jesus, Joanne; Research Institute for Tropical Medicine, Clinical Trials, Epidemiology and Biostatistics<br>Jimenez, Carolina; Médecins Sans Frontières Operational Centre Barcelona<br>Kain, Kevin; University of Toronto, Laboratory Medicine & Pathobiology<br>Suy, Keang; Cambodia Oxford Medical Research Unit, Angkor Hospital for Children; Angkor Hospital for Children<br>Koshiaris, Constantinos; University of Oxford Department of Primary Care Health Sciences, Department of Primary Health care sciences<br>Lasry, Estrella; Médecins Sans Frontières Operational Centre Barcelona<br>Mayxay, Mayfong; Lao-Oxford-Mahosot Hospital-Wellcome Trust Research Unit; Faculty of Postgraduate Studies, University of Health Sciences<br>Mondal, Dinesh; icddr,b, Centre for Nutrition and Food Security (CNFS)<br>Perera, Rafael; University of Oxford, Primary Health Care<br>Pongvongsa, Tiengkham; Lao-Oxford-Mahosot Hospital-Wellcome Trust Research Unit, Microbiology Department; Savannakhet Provincial Health Department<br>Rattanavong, Sayaphet; Lao-Oxford-Mahosot Hospital-Wellcome Trust

Research Unit, Microbiology Department
Rekart, Michael; Médecins Sans Frontières Operational Centre Barcelona
Richard-Greenblatt, Melissa; University of Pennsylvania
Shomik, Mohammad; icddr,b, Centre for Nutrition and Food Security (CNFS)
Souvannasing, Phouthalavanh; Salavan Provincial Hospital
Tallo, Veronica; Research Institute for Tropical Medicine, Clinical Trials, Epidemiology and Biostatistics
Turner, Claudia; University of Oxford, Centre for Tropical Medicine and Global Health; Angkor Hospital for Children
Turner, Paul ; University of Oxford, Centre for Tropical Medicine and Global Health; Cambodia Oxford Medical Research Unit, Angkor Hospital for Children
Waithira, Naomi; Mahidol-Oxford Tropical Medicine Research Unit, Faculty of Tropical Medicine
Watson, James A; Mahidol-Oxford Tropical Medicine Research Unit, Faculty of Tropical Medicine
Yosia, Mikhael; Médecins Sans Frontières Operational Centre Barcelona
Burza, Sakib; Médecins Sans Frontières Operational Centre Barcelona
Lubell, Yoel ; University of Oxford, Centre for Tropical Medicine and Global Health; Mahidol-Oxford Tropical Medicine Research Unit, Faculty of Tropical Medicine

Keywords: PAEDIATRICS, PRIMARY CARE, PUBLIC HEALTH, INFECTIOUS DISEASES

SCHOLARONE™
Manuscripts

I, the Submitting Author has the right to grant and does grant on behalf of all authors of the Work (as defined in the below author licence), an exclusive licence and/or a non-exclusive licence for contributions from authors who are: i) UK Crown employees; ii) where BMJ has agreed a CC-BY licence shall apply, and/or iii) in accordance with the terms applicable for US Federal Government officers or employees acting as part of their official duties; on a worldwide, perpetual, irrevocable, royalty-free basis to BMJ Publishing Group Ltd ("BMJ") its licensees and where the relevant Journal is co-owned by BMJ to the co-owners of the Journal, to publish the Work in this journal and any other BMJ products and to exploit all rights, as set out in our licence.

The Submitting Author accepts and understands that any supply made under these terms is made by BMJ to the Submitting Author unless you are acting as an employee on behalf of your employer or a postgraduate student of an affiliated institution which is paying any applicable article publishing charge ("APC") for Open Access articles. Where the Submitting Author wishes to make the Work available on an Open Access basis (and intends to pay the relevant APC), the terms of reuse of such Open Access shall be governed by a Creative Commons licence – details of these licences and which Creative Commons licence will apply to this Work are set out in our licence referred to above.

Other than as permitted in any relevant BMJ Author's Self Archiving Policies, I confirm this Work has not been accepted for publication elsewhere, is not being considered for publication elsewhere and does not duplicate material already published. I confirm all authors consent to publication of this Work and authorise the granting of this licence.

For peer review only

TITLE

Prediction of disease severity in young children presenting with acute febrile illness in resource-limited settings: a protocol for a prospective observational study

AUTHORS

Arjun Chandna,[1,2*] Endashaw M. Aderie,[3] Riris Ahmad,[4] Eggi Arguni,[4] Elizabeth A. Ashley,[2,5] Tanya Cope,[6] Vu Quoc Dat,[7] Nicholas Day,[2,6] Arjen Dondorp,[2,6] Victor Illanes,[3] Joanne de Jesus,[8] Carolina Jiménez,[3] Kevin C. Kain,[9] Suy Keang,[1,10] Constantinos Koshiaris,[11] Estrella Lasry,[3] Mayfong Mayxay,[2,5,12] Dinesh Mondal,[13] Rafael Perera,[11] Tiengkham Pongvongsa,[5,14] Sayaphet Rattanavong,[5] Michael Rekart,[3] Melissa Richard-Greenblatt,[15] Mohammad Shomik,[13] Phouthalavanh Souvannasing,[16] Veronica Tallo,[8] Claudia Turner,[2,10] Paul Turner,[1,2] Naomi Waithira,[6] James Watson,[6] Mikhael Yosia,[3] Sakib Burza[3,**] and Yoel Lubell[2,6,**]

* Corresponding author: arjun@tropmedres.ac

** These authors contributed equally

AFFILIATIONS

1. Cambodia Oxford Medical Research Unit, Angkor Hospital for Children, Siem Reap, Cambodia
2. Centre for Tropical Medicine and Global Health, University of Oxford, UK
3. *Médecins Sans Frontières* Operational Centre Barcelona
4. Centre for Tropical Medicine, Universitas Gadjah Mada, Yogyakarta, Indonesia
5. Lao-Oxford-Mahosot Hospital-Wellcome Trust Research Unit (LOMWRU), Microbiology Laboratory, Mahosot Hospital, Vientiane, Lao PDR
6. Mahidol Oxford Tropical Medicine Research Unit, Faculty of Tropical Medicine, Mahidol University, Bangkok, Thailand
7. Hanoi Medical University, Hanoi, Vietnam
8. Research Institute Tropical Medicine, Manila, The Philippines
9. Tropical Disease Unit, Department of Medicine, University of Toronto, Sandra Rotman Centre for Global Health, University Health Network-Toronto General Hospital, Canada
10. Angkor Hospital for Children, Siem Reap, Cambodia
11. Department of Primary Care Health Sciences, University of Oxford, UK

For peer review only

12. Institute of Research and Education Development, University of Health Sciences, Vientiane, Lao PDR
13. International Centre for Diarrhoeal Disease Research, Dhaka, Bangladesh
14. Savannakhet Provincial Health Department, Savannakhet Province, Lao PDR
15. University of Pennsylvania, Philadelphia, USA
16. Salavan Provincial Hospital, Salavan Province, Lao PDR

KEYWORDS

Paediatric, febrile illness, severity, sepsis, prognosis, prediction model, primary care, resource-constrained, biomarker, clinical risk score

LIST OF ABREVIATIONS

CTSG	Clinical Trials Support Group
eCRF	Electronic Case Record Form
EDTA	Ethylenediaminetetraacetic acid
iCCM	Integrated Community Case Management
IMCI	Integrated Management of Childhood Illnesses
MORU	Mahidol Oxford Tropical Medicine Research Unit
MSF	*Médecins Sans Frontières*
MSF-OCBA	*Médecins Sans Frontières* Operating Centre Barcelona
ODK	Open Data Kit
OxTREC	Oxford Tropical Research Ethics Committee
SIV	Site Initiation Visit
SOP	Standard Operating Procedure
WHO	World Health Organization

For peer review only

ABSTRACT

Introduction

In rural and difficult-to-access settings, early and accurate recognition of febrile children at risk of progressing to serious illness could contribute to improved patient outcomes and better resource allocation. This study aims to develop a prognostic clinical prediction tool to assist community healthcare providers identify febrile children who might benefit from referral or admission for facility-based medical care.

Methods and analysis

This prospective observational study will recruit at least 4,900 paediatric inpatients and outpatients under the age of five years presenting with an acute febrile illness to seven hospitals in six countries across Asia. A venous blood sample and nasopharyngeal swab is collected from each participant and detailed clinical data recorded at presentation, and each day for the first 48 hours of admission for inpatients. Multi-analyte assays are performed at reference laboratories to measure a panel of host biomarkers, as well as targeted aetiological investigations for common bacterial and viral pathogens. Clinical outcome is ascertained on Day 2 and Day 28.

Presenting syndromes, clinical outcomes and aetiology of acute febrile illness will be described and compared across sites. Following the latest guidance in prediction model building, a prognostic clinical prediction model, combining simple clinical features and measurements of host biomarkers, will be derived and geographically externally validated. The performance of the model will be evaluated in specific presenting clinical syndromes and fever aetiologies.

Ethics and dissemination

The study has received approval from all relevant international, national and institutional ethics committees. Written informed consent is provided by the caretaker of all participants. Results will be shared with local and national stakeholders, and disseminated via peer-reviewed open-access journals and scientific meetings.

Study registration

The study has been prospectively registered at ClinicalTrials.gov (26 February 2020; NCT04285021; https://clinicaltrials.gov/ct2/show/NCT04285021) and Open Science Framework (12 March 2020; DOI: 10.17605/OSF.IO/V594S).

For peer review only

ARTICLE SUMMARY

Strengths and limitations of this study

- Multi-country study to maximise generalisability of findings and enable external geographical validation of the prediction model.
- Prioritisation of simple clinical parameters and biochemical biomarkers feasible for measurement with low-cost point-of-care tests, to ensure findings are practical for resource-limited settings.
- A minimum of 12 months continuous recruitment at each study site to capture seasonal variation in febrile illness.
- Follows the latest guidance in clinical prediction research to inform sample size, sampling frame, candidate predictor selection, and derivation and validation of the clinical prediction model.
- Absence of international consensus definitions for severity of paediatric febrile illness that avoid circularity between candidate predictors and outcome categories and are practical for use in resource-limited settings; protocol-specified secondary analyses designed to address this gap.

For peer review only

INTRODUCTION

Febrile illness represents one of the most common reasons for parents to seek medical care for their children,[1-3] and a proportion progress to severe disease with substantial risk of mortality.[4-6] Distinguishing which febrile children require referral or admission to hospital from those who can safely be cared for in the community is difficult.[7] Particularly in remote, rural environments and conflict settings, referral decisions involve complex mechanisms and incur costs and risks for both patient and provider. Better assessment and prioritisation of acutely unwell children would improve patient outcomes and reduce resource misallocation.[8-10]

In resource-constrained primary care contexts the World Health Organization's (WHO) Integrated Management of Childhood Illnesses (IMCI) and Integrated Community Case Management (iCCM) guidelines are often used to assess the need for facility-based care in febrile children presenting at the community level.[11,12] However results are inconsistent,[13] adherence is poor,[14] and implementation of multiple syndrome-specific algorithms is impractical for many limited-skill health workers.[15]

Although a number of severity scores have been proposed to predict the likelihood that a febrile child might develop serious illness,[16-18] most have been evaluated in hospitalised children and hence their potential to guide admission or referral decisions remains unclear. Furthermore, many of these scores include variables that are not feasible to collect in primary care.[19] A recent systematic review concluded that the validity of existing paediatric triage tools is uncertain and that none are likely to be reliable in resource-constrained environments, with the lack of follow-up data for children not admitted highlighted as a major limitation of current research in this field.[20] Whilst some disease-specific tools have been developed,[21-23] their application is limited as it is often only possible to ascertain a microbiological cause in the minority of febrile children.

A growing body of evidence indicates that common pathophysiological pathways, reflecting endothelial injury, immune activation and coagulopathy, are shared across a spectrum of microbial aetiologies.[24-27] Microvascular dysfunction appears to occur early in the course of common childhood infections,[28] raising the possibility that markers of these pathways might provide prognostic insight. Results from a recent study in Tanzanian outpatient clinics indicate that combining measurements of markers that reflect endothelial and immune activation with simple clinical assessments could aid triage of patients presenting from the community with acute febrile illness.[29]

This multi-country, prospective study will recruit 4,900 paediatric inpatients and outpatients under the age of five years presenting with an acute febrile illness. The primary objective is to derive and geographically externally validate a prognostic clinical prediction model, combining measurements of host biomarkers and simple clinical features, to improve disease severity assessment of febrile children presenting from the community in resource-constrained settings across Asia.

For peer review only

METHODS AND ANALYSIS

Study design

This is a multi-country, observational, prospective study being conducted in Bangladesh, Cambodia, Indonesia, Laos, The Philippines and Vietnam (**Figure 1**). The study started enrolment in March 2020 and will recruit a cohort of at least 4,900 children aged between 28 days and five years presenting to hospital with acute febrile illness. Recruitment is stratified by the treating clinician's decision to admit or send home: 3,400 children whom the treating clinician decides to admit and 1,500 children sent home directly from the outpatient department.

Figure 1. Study sites*. Seven hospitals across six Asian countries where children presenting with acute febrile illness are prospectively enrolled into the study.*

Study settings

This study aims to develop a prognostic clinical prediction tool to improve assessment and prioritisation of febrile children in rural, hard-to-reach settings and decentralised models of care across Asia. However, derivation of a prediction model requires a certain number of 'outcome events' (participants who progress to develop serious illness), and hence recruiting children presenting at the most peripheral levels of the health system would be challenging. To overcome this, mid-level hospitals (equivalent to the district- or provincial-level) were selected as study sites, acknowledging that differences in care-seeking patterns between community health facilities and hospitals exist.

This compromise risks a potential loss of generalisability to community settings, the ultimate intended-use setting for the prediction tool. To mitigate this risk study sites were identified which serve as primary points of access for a predominantly rural and underserved population, the demographics of which are representative of patients presenting to lower levels of care. Hence, as far as possible, we hope to have ensured that the primary difference between the study sites and eventual intended-use sites is the frequency with which children at risk of serious illness attend, rather than systematic differences in their demographic characteristics. This will maximise the chance of successful out-of-sample validation and generalisability of the tool to community settings.

Eligibility criteria

Children within the target age range are eligible to participate if they meet all of the following inclusion criteria: 1. Their caregiver is willing and able to provide informed consent for their

For peer review only

participation; 2. They have an axillary temperature ≥ 37.5°C OR < 35.5°C OR history of fever in the last 24 hours; and 3. The onset of their illness occurred ≤ two weeks ago.

Children are not eligible to participate if they meet any of the following exclusion criteria: 1. An accident or trauma is the reason for their presentation; 2. They are presenting ≤ three days after routine immunisations; 3. They have known specific comorbidities (including, immunosuppression, active chronic infection or major cardiorespiratory conditions); 4. They have been admitted overnight at any health facility during the current illness; or 5. They have received > 15 minutes of inpatient treatment (intravenous or nebulised medications or supplemental oxygen) at the study site prior to being screened for study eligibility. To maximise diversity within the study population, participants can only be enrolled once.

Participant enrolment

At enrolment (Day 0), demographic data, perinatal and historical information and presenting clinical symptoms are collected via interview with the participant's caretaker. Anthropometric data, vital and clinical signs are measured by the research team (see below). All data is captured on electronic Case Record Forms (eCRFs) using mobile Android tablets via Open Data Kit (ODK) Collect software. A summary of study enrolment and assessment procedures is provided in the **Supporting information** (S1).

A venous blood sample is collected for batched retrospective off-site measurement of a panel of pre-specified host biomarkers (**Table 1**) and targeted aetiological investigations (**Table 2**). In addition, a nasopharyngeal swab is collected for detection of common viral pathogens. Participants are provided with routine care, as determined by the treating clinician. Blood cultures are collected when clinically indicated, processed on site (or at a nearby quality-assured laboratory) and results fed back to the treating clinical team. Where necessary, diagnostic stewardship training is provided to encourage clinically appropriate utilisation of blood cultures and assist with interpretation of antimicrobial susceptibility testing results.

Table 1. Candidate host biomarkers. *List is subject to review as new evidence comes to light during the conduct of the study.*

Table 2. Planned aetiological investigations. *Blood cultures are collected at the discretion of the treating clinician and results provided to the treating clinical team. All other aetiological investigations are performed retrospectively using standardised protocols at reference laboratories. PCR = polymerase chain reaction.*

For peer review only

Equipment for measurement of clinical parameters (pulse oximeters [Masimo Rad-5V], respiratory rate counters, weighing scales [seca 874], height/length boards, axillary thermometers and mid-upper arm circumference tapes) were procured centrally and distributed to the study sites to ensure standardisation. Data from the eCRFs are uploaded at the end of each day to a secure server located at the Mahidol-Oxford Tropical Medicine Research Unit (MORU) in Bangkok, Thailand. Prior to commencing recruitment at each site, Site Initiation Visits (SIVs) including training in the study's Standard Operating Procedures (SOPs) and ensuring the study is conducted in accordance with Good Clinical Practice (GCP), are conducted by MORU's Clinical Trials Support Group (CTSG). Monitoring is conducted at specified intervals to ensure compliance with the study protocol and perform source data verification checks.

Sample management and laboratory assessments

Participants' nasopharyngeal swabs and venous blood samples (collected in ethylenediaminetetraacetic acid [EDTA] and Fluoride-Oxalate tubes) are transported on ice to the on-site laboratory. Samples are processed within a maximum of four hours and the nasopharyngeal specimens and blood aliquots (whole blood and plasma) are stored at -20ºC for a maximum of one month before being transported on dry ice to an in-country -80ºC freezer within the vicinity of the study site. Samples are shipped on dry ice at six-monthly intervals to MORU's central reference laboratories in Bangkok, Thailand.

Multi-analyte assays will be used for quantification of host biomarkers (**Table 1**) in plasma as previously described.[49] Biomarker selection has been informed by systematic review of the available evidence,[50,51] ensuring that assays with highest likelihood of translation into clinical practice in settings similar to which the study is being conducted are prioritised. Molecular diagnostics (multiplexed PCR) will be performed on whole blood and nasopharyngeal specimens to identify common bacterial and viral causes of febrile illness (**Table 2**).

Recruitment strategy and sample frame

Participants are recruited from the outpatient and emergency departments of the study sites. Recruitment is planned over a minimum continuous 12-month period at each site to ensure seasonality is adequately captured. Recruitment reports are generated by the MORU Data Management team, disseminated to the research team and discussed at monthly data review meetings attended by the Study Management Group (including the site principal investigators, central coordinating team and study statisticians).

For peer review only

During the hours of study recruitment, all non-elective admissions of children aged between 28 days and five years are screened for eligibility. Caregivers of eligible children are asked to provide informed consent and participant enrolment is consecutive. The recruitment rate is monitored by the Study Management Group.

Children sent home directly from the hospital outpatient department are selected randomly (using random number tables) and screened for eligibility. The recruitment rate is monitored and adjusted to ensure that the recruitment period of children sent home directly from the outpatient department mirrors that for admitted children at each site.

Screening weeks

During three weeks each year, the research team screen and determine eligibility of all (or if infeasible, a representative sample of) children aged between 28 days and five years presenting to the study site during the hours of recruitment. Screening weeks are spaced throughout the year to ensure seasonal variation in patient attendance is captured. These data will be combined with the daily routinely collected hospital data to estimate the total number of eligible children presenting to the study site. This information will be used to weight the regression analysis to derive the prediction model (see *Statistical analysis*).

Participant follow-up and outcome measurement

All children are followed-up by the research team on Day 2 (window period: + 2 days) and Day 28 (window period + 7 days). Follow-up is conducted face-to-face (via return to the study site or community outreach visit) or via telephone, depending on the constraints at the different study sites. The clinical outcome of the acute febrile illness is recorded, including the details of any further care sought between enrolment and the follow-up contact. In the event that a participant is uncontactable, a minimum of two further contacts are attempted during the window period before a participant is declared lost-to-follow-up. In addition, admitted children are followed-up each day for the first two days of their admission and on the day of discharge. Information on the treatment administered by the clinical team, as well as discharge diagnosis, are extracted from the participant's medical record.

Outcome categories are ordinal (1 to 4) and calculated on Day 28 (window period: +7 days) using a composite of vital status, receipt of organ support (defined as mechanical or non-invasive ventilation, receipt of inotropic therapy or renal replacement therapy), length of inpatient hospital stay (at the study site or other health facility) and persistence of symptoms present at enrolment (**Table 3**).

For peer review only

Table 3. Outcome categories. **Organ support defined as mechanical or non-invasive ventilation, receipt of inotropic support or renal replacement therapy.*

Sample size considerations

By using a conservative estimate of R^2, a shrinkage factor of 0.9 and a prevalence of severe outcomes (outcome category 1) of 13%,[53,54] we estimated that we would need approximately 14 events per parameter for derivation of the prediction model.[55] The derivation dataset will consist of at least 3,600 children, with oversampling of those more likely to develop a severe outcome (2,400 admitted children and 1,200 children whom the treating clinician decides to send home without admission). Based on our estimated prevalence we would expect to recruit 280 children who progress to meet the primary endpoint (outcome category 1), permitting evaluation of up to 20 candidate predictors, whilst minimising the risk of over-fitting and allowing for up to a 10% attrition rate. This sample size is the minimum number of children that we aim to recruit. If feasible, we will allow for the possibility of recruiting a higher number of participants, as this will permit inclusion of more candidate predictors in the model.

The validation dataset will be geographically distinct and will consist of at least 1,000 admitted children and 300 children sent home without admission, providing a total (enriched) sample of 1,300 children. Based on the same estimated prevalence and attrition rate, this would provide us with at least the required 100 outcome events in the validation dataset.[56]

Statistical analysis

Descriptive analysis of presenting syndromes, fever aetiologies, clinical outcomes as well as candidate predictors (baseline clinical and biochemical parameters) will be carried out. Reasons for important discrepancies will be explored across sites and the derivation dataset for the prediction model will be defined. This derivation dataset will contain the outcomes (**Table 3**), baseline clinical data and host biomarkers (**Table 1**) to be included in the prediction model. Preliminary covariate selection has already been informed by subject knowledge using systematic literature review and expert judgement.[50,51,57]

Penalised ordinal logistic regression (or its multinomial equivalent if assumptions about proportionality between outcome categories are not met) will be used for further covariate selection in order to determine the final model. For continuous variables, transformations will be used if necessary. If feasible, bootstrapping will be used to estimate model performance (discrimination and calibration) and estimate the amount of optimism in the derivation dataset.

For peer review only

The final model obtained from the derivation dataset will be applied on the validation dataset and its performance will be evaluated. The c-statistic will be used to examine discrimination and calibration plots for calibration. The performance of the model at different predicted probability thresholds will be examined.

A full case analysis will be conducted if the overall amount of missing data is less than 5%. If the fraction of missing data is more than 5% then multiple imputation will be used and regression estimates will be combined using Rubin's rule. Imputation will be conducted separately for the derivation and validation datasets.

The performance of the prediction model derived in the primary analysis will be examined in children presenting with specific clinical syndromes (for example, acute respiratory infections, diarrhoeal disease or acute undifferentiated fever) and fever aetiologies. Performance will be reported using classification tables (confusion matrices) of observed probabilities against predicted probabilities.

Additional protocol-specified secondary analyses will be conducted using alternative approaches for outcome classification (for example, binary and continuous) to explore the impact on the development of the prediction model (see *Discussion*).

Ethics and dissemination

This study (protocol version 2.0; 21 January 2020) has received ethical approval from the Oxford Tropical Research Ethics Committee (OxTREC reference: 59-19), the *Médecins Sans Frontières* Institutional Review Board (MSF IRB reference: 1967) and the relevant institutional and national ethics committees of each participating country. Written, informed consent to participate (and for the storage of clinical data and biological specimens for use in future ethically-approved studies with similar aims) is obtained from the parent or legally acceptable representative of all participants.

The study protocol, standard operating procedures, data management plan, and the datasets generated and/or analysed during the current study, are freely available from the MORU and MSF Data Access Committees upon reasonable request. Results will be shared with local and national stakeholders, including the local communities at each of the study sites, and disseminated via peer-reviewed open-access journals and scientific meetings.

Patient and public involvement

Prior to finalisation of the study protocol, the concept for the research, study design and sample collection procedures were presented to the Young Persons' Advisory Group (YPAG) at the Angkor Hospital for Children (AHC), Siem Reap, Cambodia. This group, comprising around twenty

For peer review only

children aged 10 to 15 years, provided feedback on the project to ensure alignment with the priorities of the population the research is intended to benefit.

For peer review only

DISCUSSION

This prospective study will recruit at least 4,900 children across seven hospitals in six Asian countries; measure a broad panel of clinical and biochemical biomarkers; and follow participants up over an extended period to determine clinical outcome. It will then follow the latest guidance in clinical prediction model building to derive and geographically externally validate a prognostic clinical prediction model to assist community healthcare providers assess the need for facility-based medical care in children presenting with acute febrile illness in resource-constrained settings across Asia.

Despite increasing interest in clinical prediction research, many studies have limited impact.[58] This study was designed following the latest guidance:[59] the sample size calculation and recruitment strategy (sampling frame) are based on recent methodological advances,[60,61] and selection of candidate predictors was informed by expert consensus, feasibility and systematic review of the existing evidence.[50,51,57,62] Nonetheless, several aspects warrant discussion.

The eligibility criteria for this study are based around fever, yet many children, particularly younger infants, may not mount a fever in response to serious infection. By broadening the eligibility criteria to include hypothermia and *history* of fever we believe this risk will be partly mitigated. We expect to capture the majority of children with acute infectious illness and hope that 'abnormal temperature or history of fever' (rather than, for example, 'clinician-suspected infection') will provide a 'pragmatic point-of-entry' (feasible for lesser-trained community health workers) for use of the clinical prediction tool in the future.

The study will only recruit children aged between 28 days and five years, limiting our ability to develop a parsimonious model for all children presenting with suspected infection. In particular, neonates, who carry a disproportionate risk of sepsis, are excluded.[63] This decision reflects the fact that *all* febrile neonates require further assessment, and that outside the neonatal period the greatest burden occurs in children under the age of five years. Including children of all ages would have required substantially greater resources to ensure adequate power to examine the interaction of predictive performance with age.

Developing a prediction model in settings in which the outcome of interest (in this case, episodes of severe febrile illness) occurs at relatively low frequency poses unique challenges, in particular how best to obtain sufficient precision without requiring an unfeasibly large sample size.[61] Our stratified recruitment strategy 'oversamples' admitted children and provides an 'enriched' sample with more 'outcome events'. This permits evaluation of a greater number of candidate predictors, without increasing the risk of over-fitting the prediction model.[60] Triangulating study data with data from the screening weeks and routine hospital records will provide the necessary information to estimate the denominator (total number of eligible children presenting to the study sites) and weight the regression analysis to develop a prediction model applicable to lower-prevalence community settings.

For peer review only

A further challenge is the choice of outcome (reference standard). We purposively opted not to focus solely on predicting mortality. Although mortality is a 'hard' outcome, predicting death may be of limited utility, compared to predicting severe (and in many instances treatable) illness. Furthermore, mortality occurs infrequently and is influenced (mediated) by the level and quality of care (for example, the experience of the healthcare workers and access to interventions such as oxygen, fluids, antibiotics, etc.). Estimating a generalisable prediction model (generalisable outside of the studied settings) would necessitate adjustment for the correct set of mediating variables. Adjustment for mediating variables is difficult and can introduce selection bias. To avoid this issue, we have designed ordinal outcome categories, which group children according to the eventual severity of their illness, assessed throughout until Day 28.

We recognise that these categories are imperfect (for example, children may remain in hospital for longer than 48 hours for reasons other than illness severity) and outcome misclassification will underestimate the predictive performance of candidate predictors (index tests).[64] Our protocol-specified analyses are intended to explore this further. We will look to derive a prediction model using data from the subset of children with severe (outcome category 1) and non-severe (outcome category 4) illnesses only (Table 3). These binary outcome categories will be less sensitive to misclassification but may not adequately discriminate amongst more moderately unwell children.[65] We will therefore also develop and validate a pre-specified scale that quantifies illness severity on a continuum.

This study is a first step to developing a tool that a community healthcare provider could use to guide their assessment of whether a febrile child requires referral or admission for facility-based medical care. Operationalising the prediction model developed during this study will require adaptation of the algorithm to electronic and/or paper-based decision-support tools, development of low-cost point-of-care tests for any promising biomarkers for which tests do not already exist, and iterative design in partnership with community health workers and ministries of health. Implementation will need to be supported by development of health worker capacity and contextualised to the insecure contexts in which a tool like this is most urgently needed.

For peer review only

DECLARATIONS

Competing interests

The authors declare that they have no competing interests

Funding

This work is supported by MSF-OCBA and the UK Wellcome Trust (219644/Z/19/Z). MSF maintains a co-sponsor and investigator role within the study. The Wellcome Trust will have no role in the trial design, manuscript writing or decision making for publication.

Authors' contributions

Conception of the study: AC, MR, YL, SB; Contribution to study design and development of the protocol: all authors; Devised statistical analysis plan: CK, RP; Oversight of laboratory components: MRG, PT; Drafted the manuscript: AC, MY; Commented on and approved final manuscript: all authors; Principal investigators: YL and SB.

Acknowledgements

We are grateful to bioMérieux for supporting the molecular testing of the respiratory specimens.

For peer review only

REFERENCES

1. Prasad N, Sharples KJ, Murdoch DR, Crump JA. Community prevalence of fever and relationship with malaria among infants and children in low-resource areas. *Am J Trop Med Hyg* 2015; **93**(1): 178-80.
2. Fink G, D'Acremont V, Leslie HH, Cohen J. Antibiotic exposure among children younger than 5 years in low-income and middle-income countries: a cross-sectional study of nationally representative facility-based and household-based surveys. *The Lancet Infectious Diseases* 2020; **20**(2): 179-87.
3. WHO. WHO informal consultation on fever management in peripheral health care settings: a global review of evidence and practice, 2013.
4. Fleischmann-Struzek C, Goldfarb DM, Schlattmann P, Schlapbach LJ, Reinhart K, Kissoon N. The global burden of paediatric and neonatal sepsis: a systematic review. *The Lancet Respiratory Medicine* 2018; **6**(3): 223-30.
5. D'Acremont V, Kilowoko M, Kyungu E, et al. Beyond malaria--causes of fever in outpatient Tanzanian children. *N Engl J Med* 2014; **370**(9): 809-17.
6. Prasad N, Murdoch DR, Reyburn H, Crump JA. Etiology of Severe Febrile Illness in Low- and Middle-Income Countries: A Systematic Review. *PLoS One* 2015; **10**(6): e0127962.
7. McDonald CR, Weckman A, Richard-Greenblatt M, Leligdowicz A, Kain KC. Integrated fever management: disease severity markers to triage children with malaria and non-malarial febrile illness. *Malar J* 2018; **17**(1): 353.
8. Molyneux EM, Ahmad S, Robertson A. Improved triage and emergency care for children reduces inpatient mortality in a resource-constrained setting. *Bull World Health Organ* 2006; **84**(4): 314-19.
9. Achan J, Tibenderana J, Kyabayinze D, et al. Case management of severe malaria--a forgotten practice: experiences from health facilities in Uganda. *PLoS One* 2011; **6**(3): e17053.
10. Phuong HL, de Vries PJ, Nagelkerke N, et al. Acute undifferentiated fever in Binh Thuan province, Vietnam: imprecise clinical diagnosis and irrational pharmaco-therapy. *Trop Med Int Health* 2006; **11**(6): 869-79.
11. WHO. Integrated Management of Childhood Illnesses, 2014.
12. WHO. WHO/UNICEF Joint Statement - Integrated Community Case Management: an equity-focused strategy to improve access to essential treatment services for chiildren, 2012.
13. Keitel K, Kilowoko M, Kyungu E, Genton B, D'Acremont V. Performance of prediction rules and guidelines in detecting serious bacterial infections among Tanzanian febrile children. *BMC infectious diseases* 2019; **19**(1): 769.
14. Lange S, Mwisongo A, Maestad O. Why don't clinicians adhere more consistently to guidelines for the Integrated Management of Childhood Illness (IMCI)? *Soc Sci Med* 2014; **104**: 56-63.
15. Keitel K, D'Acremont V. Electronic clinical decision algorithms for the integrated primary care management of febrile children in low-resource settings: review of existing tools. *Clinical Microbiology and Infection* 2018; **24**.
16. George EC, Walker AS, Kiguli S, et al. Predicting mortality in sick African children: the FEAST Paediatric Emergency Triage (PET) Score. *BMC Med* 2015; **13**: 174.
17. Conroy AL, Hawkes M, Hayford K, et al. Prospective validation of pediatric disease severity scores to predict mortality in Ugandan children presenting with malaria and non-malaria febrile illness. *Crit Care* 2015; **19**: 47.
18. Walia S, M HM, Kumble A, Mahalingam S, Baliga BS, Nanjegowda CK. Yale Observation Scale as a Predictor of Bacteremia and Final Outcome in 3-36 Months Old Febrile Children Admitted in Tertiary Health Centres: A Hospital-Based Cross-Sectional Study. *Asian Journal of Pharmaceutical and Clinical Research* 2016; **9**(9).
19. Fung JST, Akech S, Kissoon N, Wiens MO, English M, Ansermino JM. Determining predictors of sepsis at triage among children under 5 years of age in resource-limited settings: A modified Delphi process. *PLoS One* 2019; **14**(1): e0211274.
20. Hansoti B, Jenson A, Keefe D, et al. Reliability and validity of pediatric triage tools evaluated in Low resource settings: a systematic review. *BMC Pediatr* 2017; **17**(1): 37.

For peer review only

21. Fitzgerald F, Wing K, Naveed A, et al. Development of a Pediatric Ebola Predictive Score, Sierra Leone. *Emerg Infect Dis* 2018; **24**(2): 311-9.
22. von Seidlein L, Olaosebikan R, Hendriksen IC, et al. Predicting the clinical outcome of severe falciparum malaria in african children: findings from a large randomized trial. *Clin Infect Dis* 2012; **54**(8): 1080-90.
23. Phakhounthong K, Chaovalit P, Jittamala P, et al. Predicting the severity of dengue fever in children on admission based on clinical features and laboratory indicators: application of classification tree analysis. *BMC Pediatr* 2018; **18**(1): 109.
24. Leligdowicz A, Richard-Greenblatt M, Wright J, Crowley VM, Kain KC. Endothelial Activation: The Ang/Tie Axis in Sepsis. *Front Immunol* 2018; **9**: 838.
25. Ghosh CC, David S, Zhang R, et al. Gene control of tyrosine kinase TIE2 and vascular manifestations of infections. *Proc Natl Acad Sci U S A* 2016; **113**(9): 2472-7.
26. Leligdowicz A, Conroy AL, Hawkes M, et al. Markers Of Endothelial Injury And Immune Activation Effectively Risk-Stratify Acute Febrile Syndromes In African Children. Am J Resp Crit Care Med; 2017; Washington; 2017. p. A2818.
27. Kinasewitz GT, Yan SB, Basson B, et al. Universal changes in biomarkers of coagulation and inflammation occur in patients with severe sepsis, regardless of causative micro-organism. *Crit Care* 2004; **8**(2): R82-90.
28. Charakida M, Donald AE, Terese M, et al. Endothelial dysfunction in childhood infection. *Circulation* 2005; **111**(13): 1660-5.
29. Richard-Greenblatt M, Boillat-Blanco N, Zhong K, et al. Prognostic Accuracy of Soluble Triggering Receptor Expressed on Myeloid Cells (sTREM-1)-based Algorithms in Febrile Adults Presenting to Tanzanian Outpatient Clinics. *Clin Infect Dis* 2020; **70**(7): 1304-12.
30. Conroy AL, Hawkes M, McDonald CR, et al. Host Biomarkers Are Associated With Response to Therapy and Long-Term Mortality in Pediatric Severe Malaria. *Open Forum Infect Dis* 2016; **3**(3): ofw134.
31. Lovegrove FE, Tangpukdee N, Opoka RO, et al. Serum angiopoietin-1 and -2 levels discriminate cerebral malaria from uncomplicated malaria and predict clinical outcome in African children. *PLoS One* 2009; **4**(3): e4912.
32. Yeo TW, Lampah DA, Gitawati R, et al. Angiopoietin-2 is associated with decreased endothelial nitric oxide and poor clinical outcome in severe falciparum malaria. *Proc Natl Acad Sci U S A* 2008; **105**(44): 17097-102.
33. Mankhambo LA, Banda DL, Group IPDS, et al. The role of angiogenic factors in predicting clinical outcome in severe bacterial infection in Malawian children. *Crit Care* 2010; **14**(3): R91.
34. Srikiatkhachorn A, Ajariyakhajorn C, Endy TP, et al. Virus-induced decline in soluble vascular endothelial growth receptor 2 is associated with plasma leakage in dengue hemorrhagic Fever. *J Virol* 2007; **81**(4): 1592-600.
35. Erdman LK, Dhabangi A, Musoke C, et al. Combinations of host biomarkers predict mortality among Ugandan children with severe malaria: a retrospective case-control study. *PLoS One* 2011; **6**(2): e17440.
36. Wright JK, Hayford K, Tran V, et al. Biomarkers of endothelial dysfunction predict sepsis mortality in young infants: a matched case-control study. *BMC Pediatr* 2018; **18**(1): 118.
37. Moxon CA, Chisala NV, Mzikamanda R, et al. Laboratory evidence of disseminated intravascular coagulation is associated with a fatal outcome in children with cerebral malaria despite an absence of clinically evident thrombosis or bleeding. *J Thromb Haemost* 2015; **13**(9): 1653-64.
38. Chen HL, Hung CH, Tseng HI, Yang RC. Soluble form of triggering receptor expressed on myeloid cells-1 (sTREM-1) as a diagnostic marker of serious bacterial infection in febrile infants less than three months of age. *Jpn J Infect Dis* 2008; **61**(1): 31-5.
39. Wright SW, Lovelace-Macon L, Hantrakun V, et al. sTREM-1 predicts mortality in hospitalized patients with infection in a tropical, middle-income country. *BMC Med* 2020; **18**(1): 159.
40. Singla M, Kar M, Sethi T, et al. Immune Response to Dengue Virus Infection in Pediatric Patients in New Delhi, India--Association of Viremia, Inflammatory Mediators and Monocytes with Disease Severity. *PLoS Negl Trop Dis* 2016; **10**(3): e0004497.

For peer review only

41. Fuchs A, Gotta V, Decker ML, et al. Cytokine kinetic profiles in children with acute lower respiratory tract infection: a post hoc descriptive analysis from a randomized control trial. *Clin Microbiol Infect* 2018; **24**(12): 1341 e1- e7.
42. Carrol ED, Thomson APJ, Mobbs KJ, Hart CA. The Role of RANTES in Meningococcal Disease. *J Infect Dis* 2000; **182**(1): 363-6.
43. Carrol ED, Newland P, Riordan FAI, Thomson APJ, Curtis N, Hart CA. Procalcitonin as a diagnostic marker of meningococcal disease in children presenting with fever and a rash. *Arch Dis Child* 2002; **86**: 282-5.
44. Pourakbari B, Mamishi S, Zafari J, et al. Evaluation of procalcitonin and neopterin level in serum of patients with acute bacterial infection. *Braz J Infect Dis* 2010; **14**(3): 252-5.
45. Aramburo A, Todd J, George EC, et al. Lactate clearance as a prognostic marker of mortality in severely ill febrile children in East Africa. *BMC Med* 2018; **16**(1): 37.
46. Mtove G, Nadjm B, Hendriksen IC, et al. Point-of-care measurement of blood lactate in children admitted with febrile illness to an African District Hospital. *Clin Infect Dis* 2011; **53**(6): 548-54.
47. Nadjm B, Mtove G, Amos B, et al. Blood glucose as a predictor of mortality in children admitted to the hospital with febrile illness in Tanzania. *Am J Trop Med Hyg* 2013; **89**(2): 232-7.
48. Idro R, Ndiritu M, Ogutu B, et al. Burden, Features, and Outcome of Neurological Involvement in Acute Falciparum Malaria in Kenyan Children. *JAMA* 2007; **297**(20): 2232-40.
49. Leligdowicz A, Conroy AL, Hawkes M, et al. Validation of two multiplex platforms to quantify circulating markers of inflammation and endothelial injury in severe infection. *PLoS One* 2017; **12**(4): e0175130.
50. Robinson M, Workneh M, Dittrich S, et al. Host biomarkers to predict the severity of acute febrile illness: A scoping review, 2019.
51. Chandna A, Tan R, Salim Masoud N, Lubell Y, Turner P, Keitel K. Predictors of disease severity in children presenting with acute febrile illness: a systematic review of prognostic studies. PROSPERO, 2019.
52. Leber AL, Everhart K, Daly JA, et al. Multicenter Evaluation of BioFire FilmArray Respiratory Panel 2 for Detection of Viruses and Bacteria in Nasopharyngeal Swab Samples. *J Clin Microbiol* 2018; **56**(6): e01945-17.
53. Chheng K, Carter MJ, Emary K, et al. A prospective study of the causes of febrile illness requiring hospitalization in children in Cambodia. *PLoS One* 2013; **8**(4): e60634.
54. Nijman RG, Vergouwe Y, Thompson M, et al. Clinical prediction model to aid emergency doctors managing febrile children at risk of serious bacterial infections: diagnostic study. *BMJ* 2013; **346**: f1706.
55. Riley RD, Snell KI, Ensor J, et al. Minimum sample size for developing a multivariable prediction model: PART II - binary and time-to-event outcomes. *Stat Med* 2019; **38**(7): 1276-96.
56. Collins GS, Ogundimu EO, Altman DG. Sample size considerations for the external validation of a multivariable prognostic model: a resampling study. *Stat Med* 2016; **35**(2): 214-26.
57. Li E. Guidelines for the standardized collection of predictor variables in studies for pediatric sepsis. *Scholars Portal Dataverse* 2020.
58. Bouwmeester W, Zuithoff NP, Mallett S, et al. Reporting and methods in clinical prediction research: a systematic review. *PLoS Med* 2012; **9**(5): 1-12.
59. Moons KG, Altman DG, Reitsma JB, et al. Transparent Reporting of a multivariable prediction model for Individual Prognosis or Diagnosis (TRIPOD): explanation and elaboration. *Ann Intern Med* 2015; **162**(1): W1-73.
60. Riley RD, Ensor J, Snell KIE, et al. Calculating the sample size required for developing a clinical prediction model. *BMJ* 2020; **368**: m441.
61. Holtman GA, Berger MY, Burger H, et al. Development of practical recommendations for diagnostic accuracy studies in low-prevalence situations. *J Clin Epidemiol* 2019; **114**: 38-48.
62. Royston P, Moons KG, Altman DG, Vergouwe Y. Prognosis and prognostic research: Developing a prognostic model. *BMJ* 2009; **338**: b604.
63. Rudd KE, Johnson SC, Agesa KM, et al. Global, regional, and national sepsis incidence and mortality, 1990–2017: analysis for the Global Burden of Disease Study. *The Lancet* 2020; **395**(10219): 200-11.

For peer review only

64. McHugh LC, Snyder K, Yager TD. The effect of uncertainty in patient classification on diagnostic performance estimations. *PLoS One* 2019; **14**(5): e0217146.
65. Herberg JA, Kaforou M, Wright VJ, et al. Diagnostic Test Accuracy of a 2-Transcript Host RNA Signature for Discriminating Bacterial vs Viral Infection in Febrile Children. *JAMA* 2016; **316**(8): 835-45.

For peer review only

Table 1. Candidate host biomarkers. List is subject to review as new evidence comes to light during the conduct of the study.

Host biomarker	Summary of supportive data
**Angiopoietin-1 and -2** (Ang-1 and Ang-2)	Supportive data from Asia/SSA in children/adults, that Ang-2, Ang-1 and/or Ang-2:1 ratio predicts mortality in malaria, SBI and all-cause febrile illness.[26,29-33]
**Soluble fms-like tyrosine kinase-1 and -2** (sFlt-1 and s-Flt-2)	Supportive data from SSA that sFlt-1 predicts mortality in paediatric severe malaria and adults with all-cause febrile illness;[26,29,30] in Thailand sFlt-2 discriminates uncomplicated dengue from dengue associated with plasma leak in children.[34]
**Soluble vascular adhesion molecule-1** (sVCAM-1)	Supportive data from SSA that sVCAM-1 predicts mortality in children/adults with all-cause febrile illness.[26,29]
**Soluble intercellular adhesion molecule-1** (sICAM-1)	Supportive data from Uganda that sICAM-1 predicts mortality in paediatric severe malaria and all-cause febrile illness;[26,35] in Bangladesh, sICAM-1 predicts mortality in neonatal sepsis.[36]
**Soluble tumour necrosis factor receptor-1** (sTNFR-1)	Supportive data from Tanzania that sTNFR-1 predicts mortality in children/adults with all-cause febrile illness.[26,29]
**Soluble thrombomodulin** (sTM)	Supportive data from Malawi that sTM predicts mortality in children with severe malaria.[37]
**C-X-C motif chemokine-10** (CXCL-10) / **Interferon-γ induced protein-10** (IP-10)	Supportive data from Uganda that IP-10 predicts mortality in children with severe malaria.[30]
**Soluble triggering receptor expressed on myeloid cells-1** (sTREM-1)	Supportive data from SSA that sTREM-1 predicts mortality in paediatric severe malaria and in adults/children with all-cause febrile illness;[26,29,30,35](28, 30, 32, 33) in Asia, sTREM-1 predicted length of stay in infant febrile illness and in-hospital mortality in adults hospitalised with infection.[38,39]
**Interleukin-6** (IL-6)	Supportive data from India that IL-6 is predictive of mortality in children with dengue;[40] in Switzerland, supportive data that IL-6 predicts duration of antibiotic therapy for febrile children with lower respiratory tract infections.[41]
**Interleukin-8** (IL-8)	Supportive data from India that IL-8 is predictive of mortality in children with dengue;[40] in the UK, supportive data that IL-8 predicts disease severity in children with meningococcal disease.[42]
**Interleukin-10** (IL-10)	Supportive data from India that IL-10 is predictive of mortality in children with dengue.[40]
**Chitinase-3-like protein-1** (CHI3L1)	Supportive evidence from SSA that CHI3L1 is predictive of mortality in children/adults with all-cause febrile illness.[26,29]
**Procalcitonin** (PCT)	Supportive evidence that PCT is predictive of severe illness in hospitalised children with suspected bacterial infections or meningococcal disease.[43,44]
**Lactate**	Supportive evidence that lactate is predictive of mortality in hospitalised children with febrile illness in East Africa.[45,46]
**Glucose**	Supportive evidence that glucose is predictive of mortality in hospitalised children in Tanzania and Uganda.[47]
**Haemoglobin**	Supportive evidence that haemoglobin is predictive of mortality in hospitalised children with febrile illnesses in East Africa.[46,48]
**C-reactive protein** (CRP)	Although there is limited supportive evidence for the use of CRP as a prognostic marker for disease severity, as it is the most widely studied biomarker in our region, and numerous point-of-care tests already exist, further evaluation is warranted.

For peer review only

Table 2. Planned aetiological investigations. Blood cultures are collected at the discretion of the treating clinician and results provided to the treating clinical team. All other aetiological investigations are performed retrospectively using standardised protocols at reference laboratories. PCR = polymerase chain reaction.

Pathogen	Platform	Specimen type
**Dengue virus**	PCR	Venous blood
**Chikungunya virus**	PCR	Venous blood
**Pan-Flavivirus**	PCR	Venous blood
**Pan-Alphavirus**	PCR	Venous blood
Orientia tsutsugamushi	PCR	Venous blood
***Rickettsia* spp.**	PCR	Venous blood
***Leptospira* spp.**	PCR	Venous blood
**Eubacteria (16s rDNA)**	PCR	Venous blood
**Influenza A virus**	PCR*	Nasopharyngeal swab
**Influenza B virus**	PCR*	Nasopharyngeal swab
**Respiratory Syncytial virus**	PCR*	Nasopharyngeal swab
**Bacterial bloodstream infection**	Blood culture	Venous blood

* Nasopharyngeal swab specimens will be tested using the BioFire® FilmArray® Respiratory Pathogen 2 (RP2) panel which includes a broader range of aetiological targets (www.biofiredx.com/products/the-filmarray-panels/filmarrayrp).[52] However, as causality can be difficult to determine for some of these agents, they have not all been named here.

Table 3. Outcome categories. *Organ support defined as mechanical or non-invasive ventilation, receipt of inotropic support or renal replacement therapy.

Outcome Category	Definition
1	Death or receipt of organ support* ≤ 48 hours after enrolment
2	Death > 48 hours after enrolment and before D28 AND did not meet criteria for severe disease *or*<br>Admitted for > 48 hours at any health facility before D28 AND did not meet criteria for severe disease
3	Admitted for ≤ 48 hours at any health facility before D28 AND did not meet criteria for severe or probable severe disease *or*<br>Not admitted to any health facility AND ongoing symptoms at D28
4	Not admitted to any health facility AND symptoms resolve by D28

For peer review only

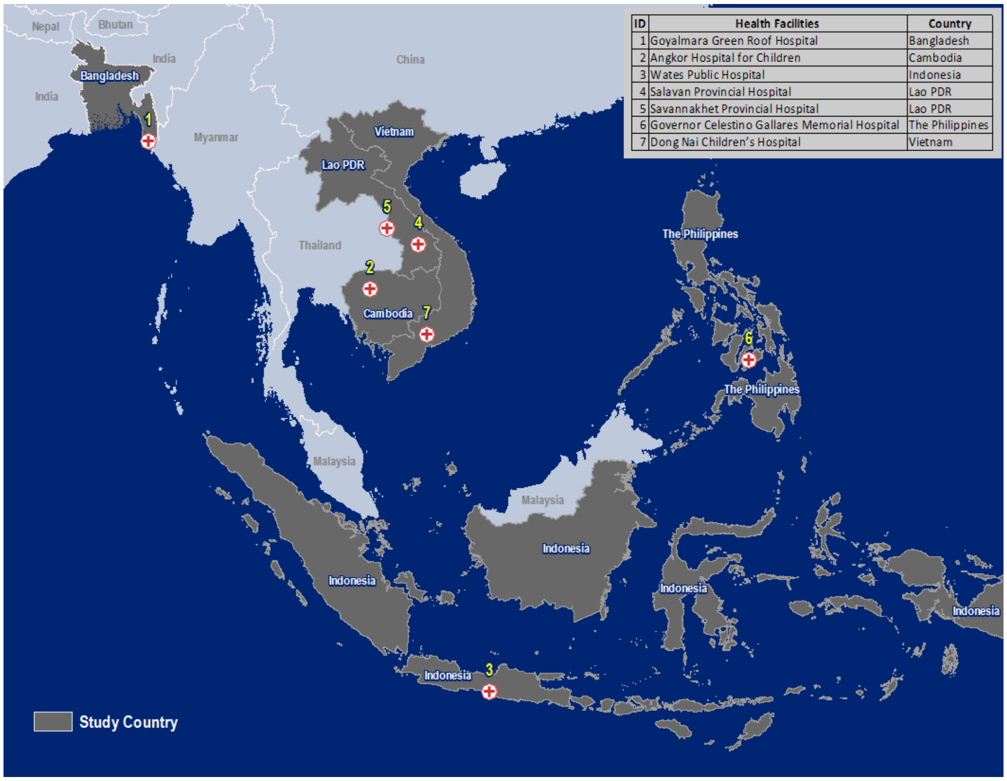

Study sites. Seven hospitals across six Asian countries where children presenting with acute febrile illness are prospectively enrolled into the study.

S1. Schedule of enrolment and assessments.

	STUDY PERIOD					
	Enrolment	Follow-up				
TIMEPOINT	DAY 0	DAY 1	DAY 2	DISCHARGE	DAY 28
**ENROLMENT**					
Eligibility screen	X				
Informed consent	X				
**ASSESSMENTS**					
Medical history	X				
Demographics	X				
Anthropometrics	X				
Clinical symptoms	X				
Clinical signs	X				
Venepuncture	X				
Nasopharyngeal swab	X				
Clinical diagnosis	•			¤	
Treatment received		¤	X	¤	X
Symptom resolution			X		X
Vital status			X	¤	X

X = all children; ¤ = admitted children only; • = children sent home directly from hospital outpatient department only

For peer review only

SPIRIT

STANDARD PROTOCOL ITEMS: RECOMMENDATIONS FOR INTERVENTIONAL TRIALS

SPIRIT 2013 Checklist: Recommended items to address in a clinical trial protocol and related documents*

Section/item	Item No	Description
**Administrative information**		
Title (Page 1)	1	**Prediction of disease severity in young children presenting with acute febrile illness in resource-limited settings**
Trial registration (Page 3)	2a	Clinicaltrials.gov NCT04285021
	2b	Date of registration: 26 February 2020<br>Secondary identifying numbers: OSF DOI 10.17605/OSF.IO/V594S<br>Source of monetary or material support: *Médecins Sans Frontières* and the UK Wellcome Trust; material support from bioMérieux<br>Primary sponsor: University of Oxford; *Médecins Sans Frontières*<br>Contact for public queries: arjun@tropmedres.ac<br>Contact for scientific queries: arjun@tropmedres.ac<br>Scientific title: Prediction of disease severity in young children presenting with acute febrile illness in resource-limited settings<br>Countries of recruitment: Bangladesh, Cambodia, Indonesia, Laos, The Philippines, Vietnam<br>Health condition studied: acute febrile illness<br>Key inclusion criteria: age > 28 days and < 5 years; axillary temperature ≥ 37.5°C OR < 35.5°C OR history of fever in the last 24 hours; onset of illness ≤ two weeks.<br>Key exclusion criteria: accident or trauma; immunisations ≤ 3 days; specific known chronic medical conditions; overnight admission during current illness; receipt of inpatient treatment > 15 minutes prior to screening.<br>Study type: prospective observational study<br>Date of first enrolment: 5 March 2020<br>Target sample size: at least 4,900 children<br>Recruitment status: recruiting<br>Primary outcome: performance of a prognostic clinical prediction model (discrimination and calibration)<br>Key secondary outcomes: description of presenting syndromes, clinical outcomes and fever aetiologies; performance of model across different presenting syndromes and fever aetiologies

For peer review only

Protocol version (Page 11)	3	Version 2.0; 21 January 2020. Version 3.0; 06 October 2020 (in submission)
Funding (Page 15)	4	Principal funding from *Médecins Sans Frontières* Operating Centre Barcelona and the UK Wellcome Trust; material support from bioMérieux for molecular testing of respiratory specimens
Roles and responsibilities (Pages 1, 15)	5a	Protocol contributors: Arjun Chandna,[1,2] Endashaw Aderie,[3] Riris Ahmad,[4] Eggi Arguni,[4] Elizabeth A. Ashley,[2,5] Tanya Cope,[6] Vu Quoc Dat,[7] Nicholas Day,[2,6] Arjen Dondorp,[2,6] Victor Illanes,[3] Joanne de Jesus,[8] Carolina Jiménez,[3] Suy Keang,[1,9] Constantinos Koshiaris,[10] Estrella Lasry,[3] Mayfong Mayxay,[2,5,11] Dinesh Mondal,[12] Rafael Perera,[10] Tiengkham Pongvongsa,[5,13] Sayaphet Rattanavong,[5] Michael Rekart,[3] Melissa Richard-Greenblatt,[14] Mohammad Shomik,[12] Veronica Tallo,[8] Claudia Turner,[2,9] Paul Turner,[1,2] Naomi Waithira,[6] James Watson,[6] Mikhael Yosia,[3] Yoel Lubell[2,6] and Sakib Burza[3]<br><br>Affiliations: 1. Cambodia Oxford Medical Research Unit, Angkor Hospital for Children, Siem Reap, Cambodia; 2. Centre for Tropical Medicine and Global Health, University of Oxford, UK; 3. *Médecins Sans Frontières* Operational Centre Barcelona Athens; 4. Centre for Tropical Medicine, Universitas Gadjah Mada, Yogyakarta, Indonesia; 5. Lao-Oxford-Mahosot Hospital-Wellcome Trust Research Unit (LOMWRU), Microbiology Laboratory, Mahosot Hospital, Vientiane, Lao PDR; 6. Mahidol Oxford Tropical Medicine Research Unit, Faculty of Tropical Medicine, Mahidol University, Bangkok, Thailand; 7. Hanoi Medical University, Hanoi, Vietnam; 8. Research Institute Tropical Medicine, Manila, The Philippines; 9. Angkor Hospital for Children, Siem Reap, Cambodia; 10. Department of Primary Care Health Sciences, University of Oxford, UK; 11. Institute of Research and Education Development, University of Health Sciences, Vientiane, Laos; 12. International Centre for Diarrhoeal Disease Research, Dhaka, Bangladesh; 13. Savannakhet Provincial Health Department, Savannakhet Province, Laos; 14. University of Pennsylvania, Philadelphia, USA<br><br>Contributions: Conception of the study: AC, MR, YL, SB; Contribution to study design and development of the protocol: all authors; Devised statistical analysis plan: CK, RP; Oversight of laboratory components: MRG, PT; Drafted the manuscript: AC, MY; Commented on and approved final manuscript: all authors.
	5b	Study sponsors: University of Oxford, tanya@tropmedres.ac; *Médecins Sans Frontières,* sakib.burza@barcelona.msf.org
	5c	MSF maintains a co-sponsor and investigator role within the study. The Wellcome Trust and bioMérieux will have no role in the trial design, manuscript writing or decision making for publication.

For peer review only

	5d	Study management group: AC, SB, YL, MY; central coordination and oversight of the study. Data management group: NW, EA, EA, SB, AC, JdJ, CK, YL, RP, MY; monitoring of recruitment rate and number of outcome events, production of recruitment reports, monitoring missing data, adjustment of recruitment strategy as required. Lead investigators: EA, EA, AC, JdJ, SR (sites) and YL, SB (overall).
**Introduction**		
Background and rationale (Page 5)	6a	In rural and difficult-to-access settings, early and accurate recognition of febrile children at risk of progressing to severe disease is challenging. Existing clinical risk scores have been developed for hospitalised children and focus on predicting mortality. There is emerging evidence that final common pathways exist across a broad range of febrile illnesses.
	6b	Not applicable: no comparators
Objectives (Page 5)	7	This study aims to identify prognostic markers in ambulatory children presenting with acute febrile illness and derive and externally validate a clinical prediction model to assist community healthcare providers identify febrile children who might benefit from referral or admission to hospital.
Trial design (Page 5)	8	Multi-country prospective observational study
**Methods: Participants, interventions, and outcomes**		
Study setting (Page 6)	9	Seven mid-level (equivalent to District or Provincial level) hospitals in Bangladesh, Cambodia, Indonesia, Laos, The Philippines and Vietnam.
Eligibility criteria (Page 6)	10	Children are eligible to participate if they meet all the following inclusion criteria: 1. Their caregiver is willing and able to provide informed consent for their participation; 2. They are aged > 28 days and < five years; 3. They have an axillary temperature ≥ 37.5°C OR < 35.5°C OR history of fever in the last 24 hours; and 4. The onset of their illness occurred ≤ two weeks ago.<br>Children are not eligible to participate if they meet any of the following exclusion criteria: 1. An accident or trauma is the reason for their presentation; 2. They are presenting ≤ three days after routine immunisations; 3. They have specific known chronic medical conditions; 4. They have been admitted overnight at any health facility during the current illness; or 5. They have received > 15 minutes of inpatient treatment (intravenous or nebulised medications or supplemental oxygen) at the study site prior to being screened for study eligibility.

For peer review only

Interventions	11a	Not applicable: no interventions
	11b	Not applicable: no interventions
	11c	Not applicable: no interventions
	11d	Children are provided with routine clinical care as determined by the treating clinicians
Outcomes (Page 9)	12	Prognostic clinical prediction model derived from a dataset containing baseline clinical variables and measurements of host biomarkers (Day 0) and clinical outcome (assessed on an ordinal scale up to Day 28). Discrimination (c-statistic) and calibration (calibration plots) of the prediction model in the external validation dataset will be reported. Performance of the model in different presenting clinical syndromes and fever aetiologies (confusion matrices) will also be reported.
Participant timeline (Pages 7-9)	13	Participants are enrolled at presentation to the study site, prior to receipt of > 15 minutes of treatment. Follow-up occurs on Day 2 and Day 28 (as well as daily for the first 48 hours of admission for inpatients).
Sample size (Page 10)	14	A minimum of 4,900 children. The sample size is based on a conservative estimate of R^2 and anticipated prevalence of severe outcomes of 13%. This will permit evaluation of up to 20 candidate predictors, whilst minimising the risk of over-fitting and allowing for up to a 10% attrition rate.
Recruitment (Pages 8-9)	15	Recruitment will be stratified according to the treating clinician's decision to admit: 3,400 admitted children and 1,500 children whom are sent home directly from the hospital's outpatient department. This will provide an enriched study population with a greater number of outcome events, permitting evaluation of more candidate predictors. Thrice yearly screening weeks are incorporated to estimate the total number of eligible children presenting to the study sites during the hours of recruitment, in order to weight the regression analysis.

Methods: Assignment of interventions (for controlled trials)

Allocation:

Sequence generation	16a	Not applicable: not an interventional study
Allocation concealment mechanism	16b	Not applicable: not an interventional study
Implementation	16c	Not applicable: not an interventional study
Blinding (masking)	17a	Not applicable: not an interventional study

	17b	Not applicable: not an interventional study

Methods: Data collection, management, and analysis

Data collection methods (Pages 7-8)	18a	Baseline clinical data is measured by dedicated research staff. Additional variables are collected via brief interview with a participant's caretaker. Research staff also collect venous blood samples for retrospective quantification of host biomarkers and aetiological investigations, and nasopharyngeal swabs for molecular testing of respiratory pathogens.<br>Equipment for measurement of clinical parameters was procured centrally and distributed to the study sites to ensure standardisation. All research staff undergo training in Good Clinical Practice, Ethical Research Practice and the study-specific Standard Operating Procedures (including clinical assessment and biological sample collection).<br>Participants are followed up on Day 2 and Day 28 (and daily for the first 48 hours of admission for inpatients). Data regarding vital status, treatment received, length of hospital stay and duration of presenting symptoms is captured and used to calculate a participant's outcome status on an ordinal severity scale.
	18b	To maximise retention follow-up can be via telephone or face-to-face (either via return to the study site or community outreach). Two telephone numbers are collected per participant and participants are reimbursed for their time and travel expenses in the case of face-to-face follow-up. A window period of 48 hours is permitted for Day 2 follow-up and 7 days for Day 28 follow-up.
Data management (Pages 7-8)	19	All data is captured using electronic Case Record Forms (eCRF) on mobile Android tablets via Open Data Kit (ODK) Collect software. Data validation activities include automated checks built into the study database to identify missing variables and flag inconsistent or invalid data during data entry. In addition, post-entry checks will be performed through profiling of the study data using statistical software.
Statistical methods (Pages 10-11)	20a	Covariate selection has already been informed by systematic review and expert judgement. Penalised ordinal logistic regression (or its multinomial equivalent if assumptions about proportionality between outcome categories are not met) will be used for further covariate selection and to derive the prediction model. Bootstrapping will be used to estimate model performance (discrimination and calibration) in the derivation dataset. The model will be applied to a geographically distinct external validation dataset and performance summarised using the c-statistic (discrimination) and calibration plots (calibration). The statistical analysis plan will be devised and published in advance of beginning the analysis.

	20b	The performance of the final model will also be reported (confusion matrices) in different presenting clinical syndromes and fever aetiologies.
	20c	A full case analysis will be conducted if the overall amount of missing data is less than 5%. If the fraction of missing data is more than 5% then multiple imputation will be used and regression estimates will be combined using Rubin's rule. Imputation will be conducted separately for the derivation and validation datasets.
**Methods: Monitoring**		
Data monitoring (Page 8)	21a	A formal data monitoring committee is not required for this observational study. A data management group (composition: NW, EA, EA, SB, AC, JdJ, CK, YL, RP, MY) is responsible for monitoring of the recruitment rate and number of outcome events, production of recruitment reports and monitoring missing data. They will make recommendations for adjustments to the recruitment strategy and target sample size as required (for example, to account for seasonal variations in patient attendances or if the prevalence of outcome events is different to that anticipated).
	21b	Not applicable: not an interventional study
Harms	22	This is an observational study. Pain will be caused due to venepuncture and mild discomfort will be caused by collection of the nasopharyngeal swab. There is a small risk that a minor bruise may be caused by traumatic venepuncture. The risks are minimised by ensuring all research staff are qualified health professionals and have undergone training in paediatric biological sample collection.
Auditing	23	Direct access will be granted to authorised representatives from the University of Oxford, *Médecins Sans Frontières*, the host institutions and the respective national ethics committees for monitoring and/or audit of the study to ensure compliance with regulations.
**Ethics and dissemination**		
Research ethics approval (Pages 3, 11)	24	This study has received ethical approval from the Oxford Tropical Research Ethics Committee (OxTREC reference: 59-19) and the *Médecins Sans Frontières* Institutional Review Board (MSF IRB reference: 1967). Ethical approval will be obtained from the national ethics committee of each participating country prior to enrolment of the first participant in that country.

Protocol amendments	25	Approval for any important protocol modifications would be sought from the relevant research ethics committees prior to implementation (unless there is an immediate and significant risk to study participants). Protocol amendments will be uploaded to the trial registries where the study is registered and attached/submitted as an additional file to any existing published versions of the protocol.
Consent or assent (Pages 3, 11)	26a	Trained research staff will seek informed written consent from the caretaker or legally appointed representative of potential participants.
	26b	Consent will be taken for the use of data (including biological samples) in future ethically-approved studies with similar aims. Participants' caretakers will be able to opt out of this clause.
Confidentiality	27	The participants will be identified only by a participant ID number on all study documents and any electronic database, with the exception of the signed consent form and the study recruitment log book, where participant/caretaker name, address and telephone details will be recorded, for the purposes of contacting participants for follow-up. All documents will be stored securely and only accessible by study staff and authorised personnel. The study will comply with the General Data Protection Regulation (GDPR), which requires that personal data must not be kept as identifiable data for longer than necessary for the purposes concerned.
Declaration of interests (Page 14)	28	The principal investigators declare that they have no competing interests.
Access to data (Page 11)	29	The datasets generated and/or analysed during the current study will be available from the MORU and MSF Data Access Committees upon reasonable request.
Ancillary and post-trial care	30	Not applicable for this observational study. If caretakers have ongoing concerns about the health of their child at the end of follow-up they will be advised to seek care from a registered medical practitioner.
Dissemination policy (Page 11)	31a	In accordance with MSF and Wellcome Trust publication policies all results generated from this study will be published in open access mode in accordance with these guidelines. The results of the study will be summarised in lay language, in both English and the language(s) commonly spoken at the study sites, and disseminated to key stakeholders and user communities.
	31b	All publications will abide by the International Committee of Medical Journal Editors recommendations of the role of authors and contributors.
	31c	The full study protocol is available at Open Science Framework (DOI: 10.17605/OSF.IO/V594S). The datasets will be available from the MORU and MSF Data Access Committees upon reasonable request.

For peer review only - http://bmjopen.bmj.com/site/about/guidelines.xhtml

Appendices

Informed consent materials	32	The consent form and participant information sheet are available on request.
Biological specimens (Pages 8, 11)	33	Any remaining sample will be archived at MORU, Thailand, in a facility with experienced personnel and appropriately monitored -80°C freezers and other relevant equipment. Samples will be made available for researchers and developers for the evaluation of new LMIC-friendly point-of-care diagnostic tests intended to guide the management of febrile patients; no time limit is set for the duration to which the samples could be stored but given the low volume and high value we anticipate they will be used up within a short time frame. Access to the archive will follow a formal process of application to the MORU and MSF Data Access Committees.

For peer review only

BMJ Open

Prediction of disease severity in young children presenting with acute febrile illness in resource-limited settings: a protocol for a prospective observational study

Journal:	*BMJ Open*
Manuscript ID	bmjopen-2020-045826.R1
Article Type:	Protocol
Date Submitted by the Author:	03-Dec-2020

Complete List of Authors:	Chandna, Arjun; Cambodia Oxford Medical Research Unit, Angkor Hospital for Children; University of Oxford, Centre for Tropical Medicine and Global Health<br>Aderie, Endashaw; Médecins Sans Frontières Operational Centre Barcelona<br>Ahmad, Riris; Universitas Gadjah Mada, Centre for Tropical Medicine<br>Arguni, Eggi; Universitas Gadjah Mada, Centre for Tropical Medicine<br>Ashley, Elizabeth ; University of Oxford, Centre for Tropical Medicine and Global Health; Lao-Oxford-Mahosot Hospital-Wellcome Trust Research Unit, Microbiology Department<br>Cope, Tanya; Mahidol-Oxford Tropical Medicine Research Unit, Faculty of Tropical Medicine<br>Dat, Vu Quoc; Hanoi Medical University<br>Day, Nicholas; Mahidol Oxford Tropical Medicine Research Unit, Faculty of Tropical Medicine; University of Oxford, Centre for Tropical Medicine and Global Health<br>Dondorp, Arjen; Mahidol-Oxford Tropical Medicine Research Unit, Faculty of Tropical Medicine; University of Oxford, Centre for Tropical Medicine and Global Health<br>Illanes, Victor; Médecins Sans Frontières Operational Centre Barcelona<br>De Jesus, Joanne; Research Institute for Tropical Medicine, Clinical Trials, Epidemiology and Biostatistics<br>Jimenez, Carolina; Médecins Sans Frontières Operational Centre Barcelona<br>Kain, Kevin; University of Toronto, Laboratory Medicine & Pathobiology<br>Suy, Keang; Cambodia Oxford Medical Research Unit, Angkor Hospital for Children; Angkor Hospital for Children<br>Koshiaris, Constantinos; University of Oxford Department of Primary Care Health Sciences, Department of Primary Health care sciences<br>Lasry, Estrella; Médecins Sans Frontières Operational Centre Barcelona<br>Mayxay, Mayfong; Lao-Oxford-Mahosot Hospital-Wellcome Trust Research Unit; Faculty of Postgraduate Studies, University of Health Sciences<br>Mondal, Dinesh; icddr,b, Centre for Nutrition and Food Security (CNFS)<br>Perera, Rafael; University of Oxford, Primary Health Care<br>Pongvongsa, Tiengkham; Lao-Oxford-Mahosot Hospital-Wellcome Trust Research Unit, Microbiology Department; Savannakhet Provincial Health Department<br>Rattanavong, Sayaphet; Lao-Oxford-Mahosot Hospital-Wellcome Trust

	Research Unit, Microbiology Department<br>Rekart, Michael; Médecins Sans Frontières Operational Centre Barcelona<br>Richard-Greenblatt, Melissa; University of Pennsylvania<br>Shomik, Mohammad; icddr,b, Centre for Nutrition and Food Security (CNFS)<br>Souvannasing, Phouthalavanh; Salavan Provincial Hospital<br>Tallo, Veronica; Research Institute for Tropical Medicine, Clinical Trials, Epidemiology and Biostatistics<br>Turner, Claudia; University of Oxford, Centre for Tropical Medicine and Global Health; Angkor Hospital for Children<br>Turner, Paul ; University of Oxford, Centre for Tropical Medicine and Global Health; Cambodia Oxford Medical Research Unit, Angkor Hospital for Children<br>Waithira, Naomi; Mahidol-Oxford Tropical Medicine Research Unit, Faculty of Tropical Medicine<br>Watson, James A; Mahidol-Oxford Tropical Medicine Research Unit, Faculty of Tropical Medicine<br>Yosia, Mikhael; Médecins Sans Frontières Operational Centre Barcelona<br>Burza, Sakib; Médecins Sans Frontières Operational Centre Barcelona<br>Lubell, Yoel ; University of Oxford, Centre for Tropical Medicine and Global Health; Mahidol-Oxford Tropical Medicine Research Unit, Faculty of Tropical Medicine

**Primary Subject Heading**:	Global health
Secondary Subject Heading:	Paediatrics, Infectious diseases, Public health
Keywords:	PAEDIATRICS, PRIMARY CARE, PUBLIC HEALTH, INFECTIOUS DISEASES

SCHOLARONE™
Manuscripts

I, the Submitting Author has the right to grant and does grant on behalf of all authors of the Work (as defined in the below author licence), an exclusive licence and/or a non-exclusive licence for contributions from authors who are: i) UK Crown employees; ii) where BMJ has agreed a CC-BY licence shall apply, and/or iii) in accordance with the terms applicable for US Federal Government officers or employees acting as part of their official duties; on a worldwide, perpetual, irrevocable, royalty-free basis to BMJ Publishing Group Ltd ("BMJ") its licensees and where the relevant Journal is co-owned by BMJ to the co-owners of the Journal, to publish the Work in this journal and any other BMJ products and to exploit all rights, as set out in our licence.

The Submitting Author accepts and understands that any supply made under these terms is made by BMJ to the Submitting Author unless you are acting as an employee on behalf of your employer or a postgraduate student of an affiliated institution which is paying any applicable article publishing charge ("APC") for Open Access articles. Where the Submitting Author wishes to make the Work available on an Open Access basis (and intends to pay the relevant APC), the terms of reuse of such Open Access shall be governed by a Creative Commons licence – details of these licences and which Creative Commons licence will apply to this Work are set out in our licence referred to above.

Other than as permitted in any relevant BMJ Author's Self Archiving Policies, I confirm this Work has not been accepted for publication elsewhere, is not being considered for publication elsewhere and does not duplicate material already published. I confirm all authors consent to publication of this Work and authorise the granting of this licence.

For peer review only

TITLE

Prediction of disease severity in young children presenting with acute febrile illness in resource-limited settings: a protocol for a prospective observational study

AUTHORS

Arjun Chandna,[1,2*] Endashaw M. Aderie,[3] Riris Ahmad,[4] Eggi Arguni,[4] Elizabeth A. Ashley,[2,5] Tanya Cope,[6] Vu Quoc Dat,[7] Nicholas Day,[2,6] Arjen Dondorp,[2,6] Victor Illanes,[3] Joanne de Jesus,[8] Carolina Jiménez,[3] Kevin C. Kain,[9] Suy Keang,[1,10] Constantinos Koshiaris,[11] Estrella Lasry,[3] Mayfong Mayxay,[2,5,12] Dinesh Mondal,[13] Rafael Perera,[11] Tiengkham Pongvongsa,[5,14] Sayaphet Rattanavong,[5] Michael Rekart,[3] Melissa Richard-Greenblatt,[15] Mohammad Shomik,[13] Phouthalavanh Souvannasing,[16] Veronica Tallo,[8] Claudia Turner,[2,10] Paul Turner,[1,2] Naomi Waithira,[6] James Watson,[6] Mikhael Yosia,[3] Sakib Burza[3,**] and Yoel Lubell[2,6,**]

* Corresponding author: arjun@tropmedres.ac

** These authors contributed equally

AFFILIATIONS

1. Cambodia Oxford Medical Research Unit, Angkor Hospital for Children, Siem Reap, Cambodia
2. Centre for Tropical Medicine and Global Health, University of Oxford, UK
3. *Médecins Sans Frontières* Operational Centre Barcelona
4. Centre for Tropical Medicine, Universitas Gadjah Mada, Yogyakarta, Indonesia
5. Lao-Oxford-Mahosot Hospital-Wellcome Trust Research Unit (LOMWRU), Microbiology Laboratory, Mahosot Hospital, Vientiane, Lao PDR
6. Mahidol Oxford Tropical Medicine Research Unit, Faculty of Tropical Medicine, Mahidol University, Bangkok, Thailand
7. Hanoi Medical University, Hanoi, Vietnam
8. Research Institute Tropical Medicine, Manila, The Philippines
9. Tropical Disease Unit, Department of Medicine, University of Toronto, Sandra Rotman Centre for Global Health, University Health Network-Toronto General Hospital, Canada
10. Angkor Hospital for Children, Siem Reap, Cambodia
11. Department of Primary Care Health Sciences, University of Oxford, UK

For peer review only - http://bmjopen.bmj.com/site/about/guidelines.xhtml

12. Institute of Research and Education Development, University of Health Sciences, Vientiane, Lao PDR
13. International Centre for Diarrhoeal Disease Research, Dhaka, Bangladesh
14. Savannakhet Provincial Health Department, Savannakhet Province, Lao PDR
15. University of Pennsylvania, Philadelphia, USA
16. Salavan Provincial Hospital, Salavan Province, Lao PDR

KEYWORDS

Paediatric, febrile illness, severity, sepsis, prognosis, prediction model, primary care, resource-constrained, biomarker, clinical risk score

LIST OF ABREVIATIONS

CTSG	Clinical Trials Support Group
eCRF	Electronic Case Record Form
EDTA	Ethylenediaminetetraacetic acid
iCCM	Integrated Community Case Management
IMCI	Integrated Management of Childhood Illnesses
MORU	Mahidol Oxford Tropical Medicine Research Unit
MSF	*Médecins Sans Frontières*
MSF-OCBA	*Médecins Sans Frontières* Operating Centre Barcelona
ODK	Open Data Kit
OxTREC	Oxford Tropical Research Ethics Committee
SIV	Site Initiation Visit
SOP	Standard Operating Procedure
WHO	World Health Organization

For peer review only

For peer review only - http://bmjopen.bmj.com/site/about/guidelines.xhtml

ABSTRACT

Introduction

In rural and difficult-to-access settings, early and accurate recognition of febrile children at risk of progressing to serious illness could contribute to improved patient outcomes and better resource allocation. This study aims to develop a prognostic clinical prediction tool to assist community healthcare providers identify febrile children who might benefit from referral or admission for facility-based medical care.

Methods and analysis

This prospective observational study will recruit at least 4,900 paediatric inpatients and outpatients under the age of five years presenting with an acute febrile illness to seven hospitals in six countries across Asia. A venous blood sample and nasopharyngeal swab is collected from each participant and detailed clinical data recorded at presentation, and each day for the first 48 hours of admission for inpatients. Multi-analyte assays are performed at reference laboratories to measure a panel of host biomarkers, as well as targeted aetiological investigations for common bacterial and viral pathogens. Clinical outcome is ascertained on Day 2 and Day 28.

Presenting syndromes, clinical outcomes and aetiology of acute febrile illness will be described and compared across sites. Following the latest guidance in prediction model building, a prognostic clinical prediction model, combining simple clinical features and measurements of host biomarkers, will be derived and geographically externally validated. The performance of the model will be evaluated in specific presenting clinical syndromes and fever aetiologies.

Ethics and dissemination

The study has received approval from all relevant international, national and institutional ethics committees. Written informed consent is provided by the caretaker of all participants. Results will be shared with local and national stakeholders, and disseminated via peer-reviewed open-access journals and scientific meetings.

Study registration

The study has been prospectively registered at ClinicalTrials.gov (26 February 2020; NCT04285021; https://clinicaltrials.gov/ct2/show/NCT04285021) and Open Science Framework (12 March 2020; DOI: 10.17605/OSF.IO/V594S).

For peer review only - http://bmjopen.bmj.com/site/about/guidelines.xhtml

ARTICLE SUMMARY

Strengths and limitations of this study

- Multi-country study with a minimum of 12 months continuous recruitment at each site to capture seasonal variation, maximise generalisability of findings and enable external geographical validation of the prediction model.
- Prioritisation of simple clinical parameters and biochemical biomarkers feasible for measurement with point-of-care tests, to ensure findings are practical for resource-limited settings.
- Follows the latest guidance in clinical prediction research to inform sample size, sampling frame, candidate predictor selection, and derivation and validation of the clinical prediction model.
- Absence of international consensus definitions for severity of paediatric febrile illness that avoid circularity between candidate predictors and outcome categories and are practical for use in resource-limited settings; protocol-specified secondary analyses designed to address this gap.
- Translation of findings will require commercialisation, availability and uptake of low-cost point-of-care tests for any promising biochemical biomarkers identified and included in the clinical prediction model.

For peer review only

INTRODUCTION

Febrile illness represents one of the most common reasons for parents to seek medical care for their children,[1-3] and a proportion progress to severe disease with substantial risk of mortality.[4-6] Distinguishing which febrile children require referral or admission to hospital from those who can safely be cared for in the community is difficult.[7] Particularly in remote, rural environments and conflict settings, referral decisions involve complex mechanisms and incur costs and risks for both patient and provider. Better assessment and prioritisation of acutely unwell children would improve patient outcomes and reduce resource misallocation.[8-10]

In resource-constrained primary care contexts the World Health Organization's (WHO) Integrated Management of Childhood Illnesses (IMCI) and Integrated Community Case Management (iCCM) guidelines are often used to assess the need for facility-based care in febrile children presenting at the community level.[11,12] However results are inconsistent,[13] adherence is poor,[14] and implementation of multiple syndrome-specific algorithms is impractical for many limited-skill health workers.[15]

Although a number of severity scores have been proposed to predict the likelihood that a febrile child might develop serious illness,[16-18] most have been evaluated in hospitalised children and hence their potential to guide admission or referral decisions remains unclear. Furthermore, many of these scores include variables that are not feasible to collect in primary care.[19] A recent systematic review concluded that the validity of existing paediatric triage tools is uncertain and that none are likely to be reliable in resource-constrained environments, with the lack of follow-up data for children not admitted highlighted as a major limitation of current research in this field.[20] Whilst some disease-specific tools have been developed,[21-23] their application is limited as it is often only possible to ascertain a microbiological cause in the minority of febrile children.

A growing body of evidence indicates that common pathophysiological pathways, reflecting endothelial injury, immune activation and coagulopathy, are shared across a spectrum of microbial aetiologies.[24-27] Microvascular dysfunction appears to occur early in the course of common childhood infections,[28] raising the possibility that markers of these pathways might provide prognostic insight. Results from a recent study in Tanzanian outpatient clinics indicate that combining measurements of markers that reflect endothelial and immune activation with simple clinical assessments could aid triage of patients presenting from the community with acute febrile illness.[29]

This multi-country, prospective study will recruit 4,900 paediatric inpatients and outpatients under the age of five years presenting with an acute febrile illness. The primary objective is to derive and geographically externally validate a prognostic clinical prediction model, combining measurements of host biomarkers and simple clinical features, to improve disease severity assessment of febrile children presenting from the community in resource-constrained settings across Asia.

For peer review only - http://bmjopen.bmj.com/site/about/guidelines.xhtml

METHODS AND ANALYSIS

Study design

This is a multi-country, observational, prospective study being conducted in Bangladesh, Cambodia, Indonesia, Laos, The Philippines and Vietnam (**Figure 1**). The study started enrolment in March 2020 and will recruit a cohort of at least 4,900 children aged between 28 days and five years presenting to hospital with acute febrile illness. Recruitment is stratified by the treating clinician's decision to admit or send home: 3,400 children whom the treating clinician decides to admit and 1,500 children sent home directly from the outpatient department.

Figure 1. Study sites*. Seven hospitals across six Asian countries where children presenting with acute febrile illness are prospectively enrolled into the study.*

Study settings

This study aims to develop a prognostic clinical prediction tool to improve assessment and prioritisation of febrile children in rural, hard-to-reach settings and decentralised models of care across Asia. However, derivation of a prediction model requires a certain number of 'outcome events' (participants who progress to develop serious illness), and hence recruiting children presenting at the most peripheral levels of the health system would be challenging. To overcome this, mid-level hospitals (equivalent to the district- or provincial-level) were selected as study sites, acknowledging that differences in care-seeking patterns between community health facilities and hospitals exist.

This compromise risks a potential loss of generalisability to community settings, the ultimate intended-use setting for the prediction tool. To mitigate this risk study sites were identified which serve as primary points of access for a predominantly rural and underserved population, the demographics of which are representative of patients presenting to lower levels of care. Hence, as far as possible, we hope to have ensured that the primary difference between the study sites and eventual intended-use sites is the frequency with which children at risk of serious illness attend, rather than systematic differences in their demographic characteristics. This will maximise the chance of successful out-of-sample validation and generalisability of the tool to community settings.

Eligibility criteria

Children within the target age range are eligible to participate if they meet all of the following inclusion criteria: 1. Their caregiver is willing and able to provide informed consent for their

For peer review only - http://bmjopen.bmj.com/site/about/guidelines.xhtml

participation; 2. They have an axillary temperature ≥ 37.5°C OR < 35.5°C OR history of fever in the last 24 hours; and 3. The onset of their illness occurred ≤ two weeks ago.

Children are not eligible to participate if they meet any of the following exclusion criteria: 1. An accident or trauma is the reason for their presentation; 2. They are presenting ≤ three days after routine immunisations; 3. They have known specific comorbidities (including, immunosuppression, active chronic infection or major cardiorespiratory conditions); 4. They have been admitted overnight at any health facility during the current illness; or 5. They have received > 15 minutes of inpatient treatment (intravenous or nebulised medications or supplemental oxygen) at the study site prior to being screened for study eligibility. To maximise diversity within the study population, participants can only be enrolled once.

Participant enrolment

At enrolment (Day 0), demographic data, perinatal and historical information and presenting clinical symptoms are collected via interview with the participant's caretaker. Anthropometric data, vital and clinical signs are measured by the research team (see below). All data is captured on electronic Case Record Forms (eCRFs) using mobile Android tablets via Open Data Kit (ODK) Collect software. A summary of study enrolment and assessment procedures is provided in the **Supporting information** (S1).

A venous blood sample is collected for batched retrospective off-site measurement of a panel of pre-specified host biomarkers (**Table 1**) and targeted aetiological investigations (**Table 2**). In addition, a nasopharyngeal swab is collected for detection of common viral pathogens. Participants are provided with routine care, as determined by the treating clinician. Blood cultures are collected when clinically indicated, processed on site (or at a nearby quality-assured laboratory) and results fed back to the treating clinical team. Where necessary, diagnostic stewardship training is provided to encourage clinically appropriate utilisation of blood cultures and assist with interpretation of antimicrobial susceptibility testing results.

For peer review only - http://bmjopen.bmj.com/site/about/guidelines.xhtml

Table 1. Candidate host biomarkers. *List is subject to review as new evidence comes to light during the conduct of the study.*

Host biomarker	Summary of supportive data
**Angiopoietin-1 and -2** (Ang-1 and Ang-2)	Supportive data from Asia/SSA in children/adults, that Ang-2, Ang-1 and/or Ang-2:1 ratio predicts mortality in malaria, SBI and all-cause febrile illness.[26,29-33]
**Soluble fms-like tyrosine kinase-1 and -2** (sFlt-1 and s-Flt-2)	Supportive data from SSA that sFlt-1 predicts mortality in paediatric severe malaria and adults with all-cause febrile illness;[26,29,30] in Thailand sFlt-2 discriminates uncomplicated dengue from dengue associated with plasma leak in children.[34]
**Soluble vascular adhesion molecule-1** (sVCAM-1)	Supportive data from SSA that sVCAM-1 predicts mortality in children/adults with all-cause febrile illness.[26,29]
**Soluble intercellular adhesion molecule-1** (sICAM-1)	Supportive data from Uganda that sICAM-1 predicts mortality in paediatric severe malaria and all-cause febrile illness;[26,35] in Bangladesh, sICAM-1 predicts mortality in neonatal sepsis.[36]
**Soluble tumour necrosis factor receptor-1** (sTNFR-1)	Supportive data from Tanzania that sTNFR-1 predicts mortality in children/adults with all-cause febrile illness.[26,29]
**Soluble thrombomodulin** (sTM)	Supportive data from Malawi that sTM predicts mortality in children with severe malaria.[37]
**C-X-C motif chemokine-10** (CXCL-10) / **Interferon-γ induced protein-10** (IP-10)	Supportive data from Uganda that IP-10 predicts mortality in children with severe malaria.[30]
**Soluble triggering receptor expressed on myeloid cells-1** (sTREM-1)	Supportive data from SSA that sTREM-1 predicts mortality in paediatric severe malaria and in adults/children with all-cause febrile illness;[26,29,30,35](28, 30, 32, 33) in Asia, sTREM-1 predicted length of stay in infant febrile illness and in-hospital mortality in adults hospitalised with infection.[38,39]
**Interleukin-6** (IL-6)	Supportive data from India that IL-6 is predictive of mortality in children with dengue;[40] in Switzerland, supportive data that IL-6 predicts duration of antibiotic therapy for febrile children with lower respiratory tract infections.[41]
**Interleukin-8** (IL-8)	Supportive data from India that IL-8 is predictive of mortality in children with dengue;[40] in the UK, supportive data that IL-8 predicts disease severity in children with meningococcal disease.[42]
**Interleukin-10** (IL-10)	Supportive data from India that IL-10 is predictive of mortality in children with dengue.[40]
**Chitinase-3-like protein-1** (CHI3L1)	Supportive evidence from SSA that CHI3L1 is predictive of mortality in children/adults with all-cause febrile illness.[26,29]
**Procalcitonin** (PCT)	Supportive evidence that PCT is predictive of severe illness in hospitalised children with suspected bacterial infections or meningococcal disease.[43,44]
**Lactate**	Supportive evidence that lactate is predictive of mortality in hospitalised children with febrile illness in East Africa.[45,46]
**Glucose**	Supportive evidence that glucose is predictive of mortality in hospitalised children in Tanzania and Uganda.[47]
**Haemoglobin**	Supportive evidence that haemoglobin is predictive of mortality in hospitalised children with febrile illnesses in East Africa.[46,48]
**C-reactive protein** (CRP)	Although there is limited supportive evidence for the use of CRP as a prognostic marker for disease severity, as it is the most widely studied biomarker in our region, and numerous point-of-care tests already exist, further evaluation is warranted.

For peer review only - http://bmjopen.bmj.com/site/about/guidelines.xhtml

Table 2. Planned aetiological investigations. *Blood cultures are collected at the discretion of the treating clinician and results provided to the treating clinical team. All other aetiological investigations are performed retrospectively using standardised protocols at reference laboratories. PCR = polymerase chain reaction.*

Pathogen	Platform	Specimen type
**Dengue virus**	PCR	Venous blood
**Chikungunya virus**	PCR	Venous blood
**Pan-Flavivirus**	PCR	Venous blood
**Pan-Alphavirus**	PCR	Venous blood
Orientia tsutsugamushi	PCR	Venous blood
***Rickettsia* spp.**	PCR	Venous blood
***Leptospira* spp.**	PCR	Venous blood
**Eubacteria (16s rDNA)**	PCR	Venous blood
**Influenza A virus**	PCR*	Nasopharyngeal swab
**Influenza B virus**	PCR*	Nasopharyngeal swab
**Respiratory Syncytial virus**	PCR*	Nasopharyngeal swab
**Bacterial bloodstream infection**	Blood culture	Venous blood

* Nasopharyngeal swab specimens will be tested using the BioFire® FilmArray® Respiratory Pathogen 2 (RP2) panel which includes a broader range of aetiological targets (www.biofiredx.com/products/the-filmarray-panels/filmarrayrp).[49] However, as causality can be difficult to determine for some of these agents, they have not all been named here.

For peer review only - http://bmjopen.bmj.com/site/about/guidelines.xhtml

Equipment for measurement of clinical parameters (pulse oximeters [Masimo Rad-5V], respiratory rate counters, weighing scales [seca 874], height/length boards, axillary thermometers and mid-upper arm circumference tapes) were procured centrally and distributed to the study sites to ensure standardisation. Data from the eCRFs are uploaded at the end of each day to a secure server located at the Mahidol-Oxford Tropical Medicine Research Unit (MORU) in Bangkok, Thailand. Prior to commencing recruitment at each site, Site Initiation Visits (SIVs) including training in the study's Standard Operating Procedures (SOPs) and ensuring the study is conducted in accordance with Good Clinical Practice (GCP), are conducted by MORU's Clinical Trials Support Group (CTSG). Monitoring is conducted at specified intervals to ensure compliance with the study protocol and perform source data verification checks.

Sample management and laboratory assessments

Participants' nasopharyngeal swabs and venous blood samples (collected in ethylenediaminetetraacetic acid [EDTA] and Fluoride-Oxalate tubes) are transported on ice to the on-site laboratory. Samples are processed within a maximum of four hours and the nasopharyngeal specimens and blood aliquots (whole blood and plasma) are stored at -20ºC for a maximum of one month before being transported on dry ice to an in-country -80ºC freezer within the vicinity of the study site. Samples are shipped on dry ice at six-monthly intervals to MORU's central reference laboratories in Bangkok, Thailand.

Multi-analyte assays will be used for quantification of host biomarkers (**Table 1**) in plasma as previously described.[50] Biomarker selection has been informed by systematic review of the available evidence,[51,52] ensuring that assays with highest likelihood of translation into clinical practice in settings similar to which the study is being conducted are prioritised. Molecular diagnostics (multiplexed PCR) will be performed on whole blood and nasopharyngeal specimens to identify common bacterial and viral causes of febrile illness (**Table 2**).

Recruitment strategy and sample frame

Participants are recruited from the outpatient and emergency departments of the study sites. Recruitment is planned over a minimum continuous 12-month period at each site to ensure seasonality is adequately captured. Recruitment reports are generated by the MORU Data Management team, disseminated to the research team and discussed at monthly data review meetings attended by the Study Management Group (including the site principal investigators, central coordinating team and study statisticians).

For peer review only - http://bmjopen.bmj.com/site/about/guidelines.xhtml

During the hours of study recruitment, all non-elective admissions of children aged between 28 days and five years are screened for eligibility. Caregivers of eligible children are asked to provide informed consent and participant enrolment is consecutive. The recruitment rate is monitored by the Study Management Group.

Children sent home directly from the hospital outpatient department are selected randomly (using random number tables) and screened for eligibility. The recruitment rate is monitored and adjusted to ensure that the recruitment period of children sent home directly from the outpatient department mirrors that for admitted children at each site.

Screening weeks

During three weeks each year, the research team screen and determine eligibility of all (or if infeasible, a representative sample of) children aged between 28 days and five years presenting to the study site during the hours of recruitment. Screening weeks are spaced throughout the year to ensure seasonal variation in patient attendance is captured. These data will be combined with the daily routinely collected hospital data to estimate the total number of eligible children presenting to the study site. This information will be used to weight the regression analysis to derive the prediction model (see *Statistical analysis*).

Participant follow-up and outcome measurement

All children are followed-up by the research team on Day 2 (window period: + 2 days) and Day 28 (window period + 7 days). Follow-up is conducted face-to-face (via return to the study site or community outreach visit) or via telephone, depending on the constraints at the different study sites. The clinical outcome of the acute febrile illness is recorded, including the details of any further care sought between enrolment and the follow-up contact. In the event that a participant is uncontactable, a minimum of two further contacts are attempted during the window period before a participant is declared lost-to-follow-up. In addition, admitted children are followed-up each day for the first two days of their admission and on the day of discharge. Information on the treatment administered by the clinical team, as well as discharge diagnosis, are extracted from the participant's medical record. A full schedule of enrolment and assessments is provided in the Supplementary File.

Outcome categories are ordinal (1 to 4) and calculated on Day 28 (window period: +7 days) using a composite of vital status, receipt of organ support (defined as mechanical or non-invasive ventilation, receipt of inotropic therapy or renal replacement therapy), length of inpatient hospital stay (at the study site or other health facility) and persistence of symptoms present at enrolment (**Table 3**).

For peer review only - http://bmjopen.bmj.com/site/about/guidelines.xhtml

Table 3. ***Outcome categories.*** **Organ support defined as mechanical or non-invasive ventilation, receipt of inotropic support or renal replacement therapy.*

Outcome Category	Definition
**1**	Death or receipt of organ support* ≤ 48 hours after enrolment
**2**	Death > 48 hours after enrolment and before D28 AND did not meet criteria for severe disease *or*<br>Admitted for > 48 hours at any health facility before D28 AND did not meet criteria for severe disease
**3**	Admitted for ≤ 48 hours at any health facility before D28 AND did not meet criteria for severe or probable severe disease *or*<br>Not admitted to any health facility AND ongoing symptoms at D28
**4**	Not admitted to any health facility AND symptoms resolve by D28

Sample size considerations

By using a conservative estimate of R^2, a shrinkage factor of 0.9 and a prevalence of severe outcomes (outcome category 1) of 13%,[53,54] we estimated that we would need approximately 14 events per parameter for derivation of the prediction model.[55] The derivation dataset will consist of at least 3,600 children, with oversampling of those more likely to develop a severe outcome (2,400 admitted children and 1,200 children whom the treating clinician decides to send home without admission). Based on our estimated prevalence we would expect to recruit 280 children who progress to meet the primary endpoint (outcome category 1), permitting evaluation of up to 20 candidate predictors, whilst minimising the risk of over-fitting and allowing for up to a 10% attrition rate. This sample size is the minimum number of children that we aim to recruit. If feasible, we will allow for the possibility of recruiting a higher number of participants, as this will permit inclusion of more candidate predictors in the model.

The validation dataset will be geographically distinct and will consist of at least 1,000 admitted children and 300 children sent home without admission, providing a total (enriched) sample of 1,300 children. Based on the same estimated prevalence and attrition rate, this would provide us with at least the required 100 outcome events in the validation dataset.[56]

Statistical analysis

For peer review only - http://bmjopen.bmj.com/site/about/guidelines.xhtml

Descriptive analysis of presenting syndromes, fever aetiologies, clinical outcomes as well as candidate predictors (baseline clinical and biochemical parameters) will be carried out. Reasons for important discrepancies will be explored across sites and the derivation dataset for the prediction model will be defined. This derivation dataset will contain the outcomes (**Table 3**), baseline clinical data and host biomarkers (**Table 1**) to be included in the prediction model. Preliminary covariate selection has already been informed by subject knowledge using systematic literature review and expert judgement.[51,52,57]

Penalised ordinal logistic regression (or its multinomial equivalent if assumptions about proportionality between outcome categories are not met) will be used for further covariate selection in order to determine the final model. For continuous variables, transformations will be used if necessary. If feasible, bootstrapping will be used to estimate model performance (discrimination and calibration) and estimate the amount of optimism in the derivation dataset.

The final model obtained from the derivation dataset will be applied on the validation dataset and its performance will be evaluated. The c-statistic will be used to examine discrimination and calibration plots for calibration. The performance of the model at different predicted probability thresholds will be examined.

A full case analysis will be conducted if the overall amount of missing data is less than 5%. If the fraction of missing data is more than 5% then multiple imputation will be used and regression estimates will be combined using Rubin's rule. Imputation will be conducted separately for the derivation and validation datasets.

The performance of the prediction model derived in the primary analysis will be examined in children presenting with specific clinical syndromes (for example, acute respiratory infections, diarrhoeal disease or acute undifferentiated fever) and fever aetiologies. Performance will be reported using classification tables (confusion matrices) of observed probabilities against predicted probabilities.

Additional protocol-specified secondary analyses will be conducted using alternative approaches for outcome classification (for example, binary and continuous) to explore the impact on the development of the prediction model (see *Discussion*).

Patient and public involvement

Prior to finalisation of the study protocol, the concept for the research, study design and sample collection procedures were presented to the Young Persons' Advisory Group (YPAG) at the Angkor Hospital for Children (AHC), Siem Reap, Cambodia. This group, comprising around twenty children aged 10 to 15 years, provided feedback on the project to ensure alignment with the priorities of the population the research is intended to benefit.

For peer review only - http://bmjopen.bmj.com/site/about/guidelines.xhtml

DISCUSSION

This prospective study will recruit at least 4,900 children across seven hospitals in six Asian countries; measure a broad panel of clinical and biochemical biomarkers; and follow participants up over an extended period to determine clinical outcome. It will then follow the latest guidance in clinical prediction model building to derive and geographically externally validate a prognostic clinical prediction model to assist community healthcare providers assess the need for facility-based medical care in children presenting with acute febrile illness in resource-constrained settings across Asia.

Despite increasing interest in clinical prediction research, many studies have limited impact.[58] This study was designed following the latest guidance:[59] the sample size calculation and recruitment strategy (sampling frame) are based on recent methodological advances,[60,61] and selection of candidate predictors was informed by expert consensus, feasibility and systematic review of the existing evidence.[51,52,57,62] Nonetheless, several aspects warrant discussion.

The eligibility criteria for this study are based around fever, yet many children, particularly younger infants, may not mount a fever in response to serious infection. By broadening the eligibility criteria to include hypothermia and *history* of fever we believe this risk will be partly mitigated. We expect to capture the majority of children with acute infectious illness and hope that 'abnormal temperature or history of fever' (rather than, for example, 'clinician-suspected infection') will provide a 'pragmatic point-of-entry' (feasible for lesser-trained community health workers) for use of the clinical prediction tool in the future.

The study will only recruit children aged between 28 days and five years, limiting our ability to develop a parsimonious model for all children presenting with suspected infection. In particular, neonates, who carry a disproportionate risk of sepsis, are excluded.[63] This decision reflects the fact that *all* febrile neonates require further assessment, and that outside the neonatal period the greatest burden occurs in children under the age of five years. Including children of all ages would have required substantially greater resources to ensure adequate power to examine the interaction of predictive performance with age.

Developing a prediction model in settings in which the outcome of interest (in this case, episodes of severe febrile illness) occurs at relatively low frequency poses unique challenges, in particular how best to obtain sufficient precision without requiring an unfeasibly large sample size.[61] Our stratified recruitment strategy 'oversamples' admitted children and provides an 'enriched' sample with more 'outcome events'. This permits evaluation of a greater number of candidate predictors, without increasing the risk of over-fitting the prediction model.[60] Triangulating study data with data from the screening weeks and routine hospital records will provide the necessary information to estimate the denominator (total number of eligible children presenting to the study sites) and weight the regression analysis to develop a prediction model applicable to lower-prevalence community settings.

For peer review only - http://bmjopen.bmj.com/site/about/guidelines.xhtml

A further challenge is the choice of outcome (reference standard). We purposively opted not to focus solely on predicting mortality. Although mortality is a 'hard' outcome, predicting death may be of limited utility, compared to predicting severe (and in many instances treatable) illness. Furthermore, mortality occurs infrequently and is influenced (mediated) by the level and quality of care (for example, the experience of the healthcare workers and access to interventions such as oxygen, fluids, antibiotics, etc.). Estimating a generalisable prediction model (generalisable outside of the studied settings) would necessitate adjustment for the correct set of mediating variables. Adjustment for mediating variables is difficult and can introduce selection bias. To avoid this issue, we have designed ordinal outcome categories, which group children according to the eventual severity of their illness, assessed throughout until Day 28.

We recognise that these categories are imperfect (for example, children may remain in hospital for longer than 48 hours for reasons other than illness severity) and outcome misclassification will underestimate the predictive performance of candidate predictors (index tests).[64] Our protocol-specified analyses are intended to explore this further. We will look to derive a prediction model using data from the subset of children with severe (outcome category 1) and non-severe (outcome category 4) illnesses only (Table 3). These binary outcome categories will be less sensitive to misclassification but may not adequately discriminate amongst more moderately unwell children.[65] We will therefore also develop and validate a pre-specified scale that quantifies illness severity on a continuum.

This study is a first step to developing a tool that a community healthcare provider could use to guide their assessment of whether a febrile child requires referral or admission for facility-based medical care. Operationalising the prediction model developed during this study will require adaptation of the algorithm to electronic and/or paper-based decision-support tools, development of low-cost point-of-care tests for any promising biomarkers for which tests do not already exist, and iterative design in partnership with community health workers and ministries of health. Implementation will need to be supported by development of health worker capacity and contextualised to the insecure contexts in which a tool like this is most urgently needed.

For peer review only - http://bmjopen.bmj.com/site/about/guidelines.xhtml

ETHICS AND DISSEMINATION

This study (protocol version 2.0; 21 January 2020) has received ethical approval from the Oxford Tropical Research Ethics Committee (OxTREC reference: 59-19), the *Médecins Sans Frontières* Institutional Review Board (MSF IRB reference: 1967) and the relevant institutional and national ethics committees of each participating country. Any necessary protocol amendments will be approved by these same ethics committees prior to implementation. Written, informed consent to participate (and for the storage of clinical data and biological specimens for use in future ethically-approved studies with similar aims) is obtained from the parent or legally acceptable representative of all participants.

The study protocol, informed consent materials, standard operating procedures, data management plan, and the datasets generated and/or analysed during the current study, are freely available from the MORU and MSF Data Access Committees upon reasonable request. Results will be shared with local and national stakeholders, including the local communities at each of the study sites, and disseminated via peer-reviewed open-access journals and scientific meetings.

For peer review only

For peer review only - http://bmjopen.bmj.com/site/about/guidelines.xhtml

DECLARATIONS

Competing interests

The authors declare that they have no competing interests

Funding

This work is supported by MSF-OCBA and the UK Wellcome Trust (219644/Z/19/Z) and co-sponsored by the University of Oxford and MSF-OCBA. MSF-OCBA maintains a co-sponsor and investigator role within the study. The Wellcome Trust will have no role in the trial design, manuscript writing or decision making for publication.

Authors' contributions

Conception of the study: AC, MR, SB, YL; Contribution to study design and development of the protocol: AC, EMA, RA, EA, EAA, TC, VQD, ND, AD, VI, JdJ, CJ, KCK, SK, CK, EL, MM, DM, RP, TP, SR. MR, MRG, MS, PS, VT, CT, PT, NW, JW, MY, SB, YL; Devised statistical analysis plan: CK, RP; Oversight of laboratory components: MRG, PT; Drafted the manuscript: AC, MY; Commented on and approved final manuscript: AC, EMA, RA, EA, EAA, TC, VQD, ND, AD, VI, JdJ, CJ, KCK, SK, CK, EL, MM, DM, RP, TP, SR. MR, MRG, MS, PS, VT, CT, PT, NW, JW, MY, SB, YL; Principal investigators: SB and YL.

Acknowledgements

We are grateful to bioMérieux for supporting the molecular testing of the respiratory specimens.

For peer review only

REFERENCES

1. Prasad N, Sharples KJ, Murdoch DR, Crump JA. Community prevalence of fever and relationship with malaria among infants and children in low-resource areas. *Am J Trop Med Hyg* 2015; **93**(1): 178-80.
2. Fink G, D'Acremont V, Leslie HH, Cohen J. Antibiotic exposure among children younger than 5 years in low-income and middle-income countries: a cross-sectional study of nationally representative facility-based and household-based surveys. *The Lancet Infectious Diseases* 2020; **20**(2): 179-87.
3. World Health Organization. Informal consultation on fever management in peripheral health care settings: a global review of evidence and practice. Geneva, 2013.
4. Fleischmann-Struzek C, Goldfarb DM, Schlattmann P, Schlapbach LJ, Reinhart K, Kissoon N. The global burden of paediatric and neonatal sepsis: a systematic review. *The Lancet Respiratory Medicine* 2018; **6**(3): 223-30.
5. D'Acremont V, Kilowoko M, Kyungu E, et al. Beyond malaria--causes of fever in outpatient Tanzanian children. *N Engl J Med* 2014; **370**(9): 809-17.
6. Prasad N, Murdoch DR, Reyburn H, Crump JA. Etiology of Severe Febrile Illness in Low- and Middle-Income Countries: A Systematic Review. *PLoS One* 2015; **10**(6): e0127962.
7. McDonald CR, Weckman A, Richard-Greenblatt M, Leligdowicz A, Kain KC. Integrated fever management: disease severity markers to triage children with malaria and non-malarial febrile illness. *Malar J* 2018; **17**(1): 353.
8. Molyneux EM, Ahmad S, Robertson A. Improved triage and emergency care for children reduces inpatient mortality in a resource-constrained setting. *Bull World Health Organ* 2006; **84**(4): 314-19.
9. Achan J, Tibenderana J, Kyabayinze D, et al. Case management of severe malaria--a forgotten practice: experiences from health facilities in Uganda. *PLoS One* 2011; **6**(3): e17053.
10. Phuong HL, de Vries PJ, Nagelkerke N, et al. Acute undifferentiated fever in Binh Thuan province, Vietnam: imprecise clinical diagnosis and irrational pharmaco-therapy. *Trop Med Int Health* 2006; **11**(6): 869-79.
11. World Health Organization. Integrated Management of Childhood Illnesses, 2014.
12. World Health Organization. WHO/UNICEF Joint Statement - Integrated Community Case Management: an equity-focused strategy to improve access to essential treatment services for chiildren, 2012.
13. Keitel K, Kilowoko M, Kyungu E, Genton B, D'Acremont V. Performance of prediction rules and guidelines in detecting serious bacterial infections among Tanzanian febrile children. *BMC infectious diseases* 2019; **19**(1): 769.
14. Lange S, Mwisongo A, Maestad O. Why don't clinicians adhere more consistently to guidelines for the Integrated Management of Childhood Illness (IMCI)? *Soc Sci Med* 2014; **104**: 56-63.
15. Keitel K, D'Acremont V. Electronic clinical decision algorithms for the integrated primary care management of febrile children in low-resource settings: review of existing tools. *Clinical Microbiology and Infection* 2018; **24**.
16. George EC, Walker AS, Kiguli S, et al. Predicting mortality in sick African children: the FEAST Paediatric Emergency Triage (PET) Score. *BMC Med* 2015; **13**: 174.

For peer review only - http://bmjopen.bmj.com/site/about/guidelines.xhtml

17. Conroy AL, Hawkes M, Hayford K, et al. Prospective validation of pediatric disease severity scores to predict mortality in Ugandan children presenting with malaria and non-malaria febrile illness. *Crit Care* 2015; **19**: 47.
18. Walia S, M HM, Kumble A, Mahalingam S, Baliga BS, Nanjegowda CK. Yale Observation Scale as a Predictor of Bacteremia and Final Outcome in 3-36 Months Old Febrile Children Admitted in Tertiary Health Centres: A Hospital-Based Cross-Sectional Study. *Asian Journal of Pharmaceutical and Clinical Research* 2016; **9**(9).
19. Fung JST, Akech S, Kissoon N, Wiens MO, English M, Ansermino JM. Determining predictors of sepsis at triage among children under 5 years of age in resource-limited settings: A modified Delphi process. *PLoS One* 2019; **14**(1): e0211274.
20. Hansoti B, Jenson A, Keefe D, et al. Reliability and validity of pediatric triage tools evaluated in Low resource settings: a systematic review. *BMC Pediatr* 2017; **17**(1): 37.
21. Fitzgerald F, Wing K, Naveed A, et al. Development of a Pediatric Ebola Predictive Score, Sierra Leone. *Emerg Infect Dis* 2018; **24**(2): 311-9.
22. von Seidlein L, Olaosebikan R, Hendriksen IC, et al. Predicting the clinical outcome of severe falciparum malaria in african children: findings from a large randomized trial. *Clin Infect Dis* 2012; **54**(8): 1080-90.
23. Phakhounthong K, Chaovalit P, Jittamala P, et al. Predicting the severity of dengue fever in children on admission based on clinical features and laboratory indicators: application of classification tree analysis. *BMC Pediatr* 2018; **18**(1): 109.
24. Leligdowicz A, Richard-Greenblatt M, Wright J, Crowley VM, Kain KC. Endothelial Activation: The Ang/Tie Axis in Sepsis. *Front Immunol* 2018; **9**: 838.
25. Ghosh CC, David S, Zhang R, et al. Gene control of tyrosine kinase TIE2 and vascular manifestations of infections. *Proc Natl Acad Sci U S A* 2016; **113**(9): 2472-7.
26. Leligdowicz A, Conroy AL, Hawkes M, et al. Markers Of Endothelial Injury And Immune Activation Effectively Risk-Stratify Acute Febrile Syndromes In African Children. Am J Resp Crit Care Med; 2017; Washington; 2017. p. A2818.
27. Kinasewitz GT, Yan SB, Basson B, et al. Universal changes in biomarkers of coagulation and inflammation occur in patients with severe sepsis, regardless of causative micro-organism. *Crit Care* 2004; **8**(2): R82-90.
28. Charakida M, Donald AE, Terese M, et al. Endothelial dysfunction in childhood infection. *Circulation* 2005; **111**(13): 1660-5.
29. Richard-Greenblatt M, Boillat-Blanco N, Zhong K, et al. Prognostic Accuracy of Soluble Triggering Receptor Expressed on Myeloid Cells (sTREM-1)-based Algorithms in Febrile Adults Presenting to Tanzanian Outpatient Clinics. *Clin Infect Dis* 2020; **70**(7): 1304-12.
30. Conroy AL, Hawkes M, McDonald CR, et al. Host Biomarkers Are Associated With Response to Therapy and Long-Term Mortality in Pediatric Severe Malaria. *Open Forum Infect Dis* 2016; **3**(3): ofw134.
31. Lovegrove FE, Tangpukdee N, Opoka RO, et al. Serum angiopoietin-1 and -2 levels discriminate cerebral malaria from uncomplicated malaria and predict clinical outcome in African children. *PLoS One* 2009; **4**(3): e4912.
32. Yeo TW, Lampah DA, Gitawati R, et al. Angiopoietin-2 is associated with decreased endothelial nitric oxide and poor clinical outcome in severe falciparum malaria. *Proc Natl Acad Sci U S A* 2008; **105**(44): 17097-102.

For peer review only - http://bmjopen.bmj.com/site/about/guidelines.xhtml

33. Mankhambo LA, Banda DL, Group IPDS, et al. The role of angiogenic factors in predicting clinical outcome in severe bacterial infection in Malawian children. *Crit Care* 2010; **14**(3): R91.
34. Srikiatkhachorn A, Ajariyakhajorn C, Endy TP, et al. Virus-induced decline in soluble vascular endothelial growth receptor 2 is associated with plasma leakage in dengue hemorrhagic Fever. *J Virol* 2007; **81**(4): 1592-600.
35. Erdman LK, Dhabangi A, Musoke C, et al. Combinations of host biomarkers predict mortality among Ugandan children with severe malaria: a retrospective case-control study. *PLoS One* 2011; **6**(2): e17440.
36. Wright JK, Hayford K, Tran V, et al. Biomarkers of endothelial dysfunction predict sepsis mortality in young infants: a matched case-control study. *BMC Pediatr* 2018; **18**(1): 118.
37. Moxon CA, Chisala NV, Mzikamanda R, et al. Laboratory evidence of disseminated intravascular coagulation is associated with a fatal outcome in children with cerebral malaria despite an absence of clinically evident thrombosis or bleeding. *J Thromb Haemost* 2015; **13**(9): 1653-64.
38. Chen HL, Hung CH, Tseng HI, Yang RC. Soluble form of triggering receptor expressed on myeloid cells-1 (sTREM-1) as a diagnostic marker of serious bacterial infection in febrile infants less than three months of age. *Jpn J Infect Dis* 2008; **61**(1): 31-5.
39. Wright SW, Lovelace-Macon L, Hantrakun V, et al. sTREM-1 predicts mortality in hospitalized patients with infection in a tropical, middle-income country. *BMC Med* 2020; **18**(1): 159.
40. Singla M, Kar M, Sethi T, et al. Immune Response to Dengue Virus Infection in Pediatric Patients in New Delhi, India--Association of Viremia, Inflammatory Mediators and Monocytes with Disease Severity. *PLoS Negl Trop Dis* 2016; **10**(3): e0004497.
41. Fuchs A, Gotta V, Decker ML, et al. Cytokine kinetic profiles in children with acute lower respiratory tract infection: a post hoc descriptive analysis from a randomized control trial. *Clin Microbiol Infect* 2018; **24**(12): 1341 e1- e7.
42. Carrol ED, Thomson APJ, Mobbs KJ, Hart CA. The Role of RANTES in Meningococcal Disease. *J Infect Dis* 2000; **182**(1): 363-6.
43. Carrol ED, Newland P, Riordan FAI, Thomson APJ, Curtis N, Hart CA. Procalcitonin as a diagnostic marker of meningococcal disease in children presenting with fever and a rash. *Arch Dis Child* 2002; **86**: 282-5.
44. Pourakbari B, Mamishi S, Zafari J, et al. Evaluation of procalcitonin and neopterin level in serum of patients with acute bacterial infection. *Braz J Infect Dis* 2010; **14**(3): 252-5.
45. Aramburo A, Todd J, George EC, et al. Lactate clearance as a prognostic marker of mortality in severely ill febrile children in East Africa. *BMC Med* 2018; **16**(1): 37.
46. Mtove G, Nadjm B, Hendriksen IC, et al. Point-of-care measurement of blood lactate in children admitted with febrile illness to an African District Hospital. *Clin Infect Dis* 2011; **53**(6): 548-54.
47. Nadjm B, Mtove G, Amos B, et al. Blood glucose as a predictor of mortality in children admitted to the hospital with febrile illness in Tanzania. *Am J Trop Med Hyg* 2013; **89**(2): 232-7.
48. Idro R, Ndiritu M, Ogutu B, et al. Burden, Features, and Outcome of Neurological Involvement in Acute Falciparum Malaria in Kenyan Children. *JAMA* 2007; **297**(20): 2232-40.

For peer review only

For peer review only - http://bmjopen.bmj.com/site/about/guidelines.xhtml

49. Leber AL, Everhart K, Daly JA, et al. Multicenter Evaluation of BioFire FilmArray Respiratory Panel 2 for Detection of Viruses and Bacteria in Nasopharyngeal Swab Samples. *J Clin Microbiol* 2018; **56**(6): e01945-17.
50. Leligdowicz A, Conroy AL, Hawkes M, et al. Validation of two multiplex platforms to quantify circulating markers of inflammation and endothelial injury in severe infection. *PLoS One* 2017; **12**(4): e0175130.
51. Robinson M, Workneh M, Dittrich S, et al. Host biomarkers to predict the severity of acute febrile illness: A scoping review, 2019.
52. Chandna A, Tan R, Salim Masoud N, Lubell Y, Turner P, Keitel K. Predictors of disease severity in children presenting with acute febrile illness: a systematic review of prognostic studies. PROSPERO, 2019.
53. Chheng K, Carter MJ, Emary K, et al. A prospective study of the causes of febrile illness requiring hospitalization in children in Cambodia. *PLoS One* 2013; **8**(4): e60634.
54. Nijman RG, Vergouwe Y, Thompson M, et al. Clinical prediction model to aid emergency doctors managing febrile children at risk of serious bacterial infections: diagnostic study. *BMJ* 2013; **346**: f1706.
55. Riley RD, Snell KI, Ensor J, et al. Minimum sample size for developing a multivariable prediction model: PART II - binary and time-to-event outcomes. *Stat Med* 2019; **38**(7): 1276-96.
56. Collins GS, Ogundimu EO, Altman DG. Sample size considerations for the external validation of a multivariable prognostic model: a resampling study. *Stat Med* 2016; **35**(2): 214-26.
57. Li E. Guidelines for the standardized collection of predictor variables in studies for pediatric sepsis. *Scholars Portal Dataverse* 2020.
58. Bouwmeester W, Zuithoff NP, Mallett S, et al. Reporting and methods in clinical prediction research: a systematic review. *PLoS Med* 2012; **9**(5): 1-12.
59. Moons KG, Altman DG, Reitsma JB, et al. Transparent Reporting of a multivariable prediction model for Individual Prognosis or Diagnosis (TRIPOD): explanation and elaboration. *Ann Intern Med* 2015; **162**(1): W1-73.
60. Riley RD, Ensor J, Snell KIE, et al. Calculating the sample size required for developing a clinical prediction model. *BMJ* 2020; **368**: m441.
61. Holtman GA, Berger MY, Burger H, et al. Development of practical recommendations for diagnostic accuracy studies in low-prevalence situations. *J Clin Epidemiol* 2019; **114**: 38-48.
62. Royston P, Moons KG, Altman DG, Vergouwe Y. Prognosis and prognostic research: Developing a prognostic model. *BMJ* 2009; **338**: b604.
63. Rudd KE, Johnson SC, Agesa KM, et al. Global, regional, and national sepsis incidence and mortality, 1990–2017: analysis for the Global Burden of Disease Study. *The Lancet* 2020; **395**(10219): 200-11.
64. McHugh LC, Snyder K, Yager TD. The effect of uncertainty in patient classification on diagnostic performance estimations. *PLoS One* 2019; **14**(5): e0217146.
65. Herberg JA, Kaforou M, Wright VJ, et al. Diagnostic Test Accuracy of a 2-Transcript Host RNA Signature for Discriminating Bacterial vs Viral Infection in Febrile Children. *JAMA* 2016; **316**(8): 835-45.

For peer review only - http://bmjopen.bmj.com/site/about/guidelines.xhtml

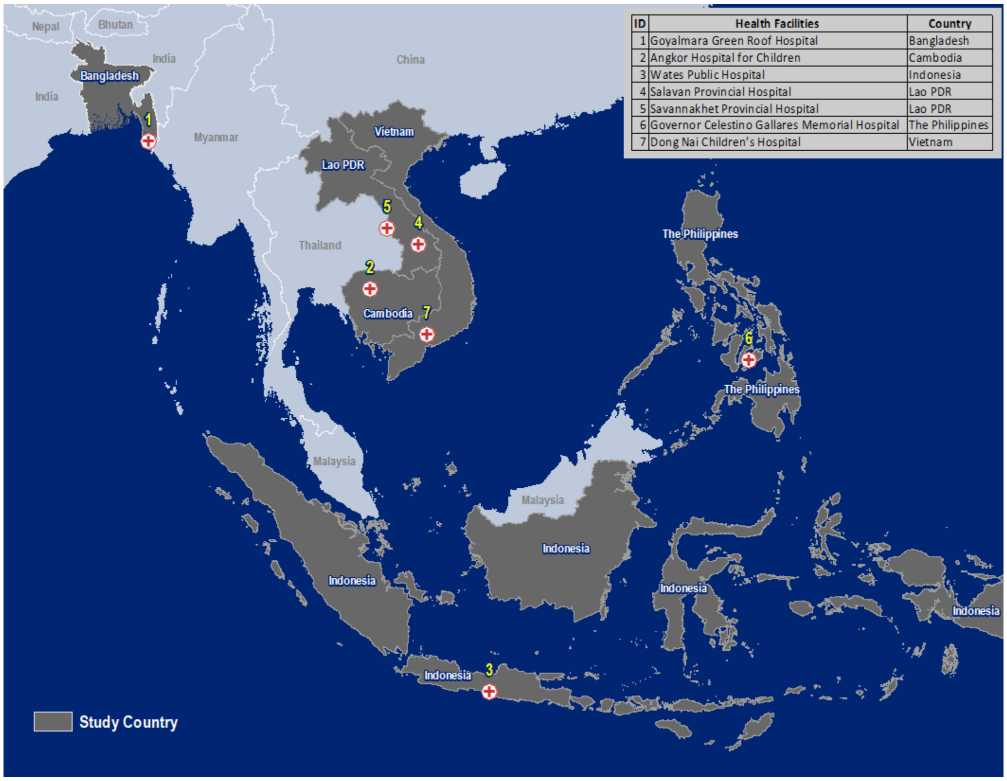

Study sites. Seven hospitals across six Asian countries where children presenting with acute febrile illness are prospectively enrolled into the study.

S1. Schedule of enrolment and assessments.

	STUDY PERIOD				
	Enrolment	Follow-up			
TIMEPOINT	DAY 0	DAY 1	DAY 2	DISCHARGE	DAY 28
**ENROLMENT**					
Eligibility screen	X				
Informed consent	X				
**ASSESSMENTS**					
Medical history	X				
Demographics	X				
Anthropometrics	X				
Clinical symptoms	X				
Clinical signs	X				
Venepuncture	X				
Nasopharyngeal swab	X				
Clinical diagnosis	•			¤	
Treatment received		¤	X	¤	X
Symptom resolution			X		X
Vital status			X	¤	X

X = all children; ¤ = admitted children only; • = children sent home directly from hospital outpatient department only

For peer review only

STANDARD PROTOCOL ITEMS: RECOMMENDATIONS FOR INTERVENTIONAL TRIALS

SPIRIT 2013 Checklist: Recommended items to address in a clinical trial protocol and related documents*

Section/item	Item No	Description
**Administrative information**		
Title	1	Page 1
Trial registration	2a	Page 3
	2b	Date of registration: Page 3<br>Secondary identifying numbers: Page 3<br>Source of monetary or material support: Page 15<br>Primary sponsor: Page 15<br>Contact for public queries: Page 1<br>Contact for scientific queries: Page 1<br>Scientific title: Page 1<br>Countries of recruitment: Page 6<br>Health condition studied: Page 6<br>Key inclusion criteria: Pages 6-7<br>Key exclusion criteria: Page 7<br>Study type: Page 6<br>Date of first enrolment: Page 6<br>Target sample size: Page 6<br>Recruitment status: Page 6<br>Primary outcome: Pages 9-10<br>Key secondary outcomes: Page 11
Protocol version	3	Page 14
Funding	4	Page 15
Roles and responsibilities	5a	Protocol contributors: Page 1<br>Contributions: Page 15
	5b	Study sponsors: Page 15
	5c	Role of study sponsor: Page 15
	5d	Study management group: Pages 8-9

For peer review only

Introduction

Background and rationale	6a	Page 5
	6b	Not applicable
Objectives	7	Page 5
Trial design	8	Page 5

Methods: Participants, interventions, and outcomes

Study setting	9	Page 6
Eligibility criteria	10	Pages 6-7
Interventions	11a	Not applicable
	11b	Not applicable
	11c	Not applicable
	11d	Page 7
Outcomes	12	Pages 9-10
Participant timeline	13	Pages 7-9
Sample size	14	Page 10
Recruitment	15	Pages 8-9

Methods: Assignment of interventions (for controlled trials)

Allocation:		
Sequence generation	16a	Not applicable
Allocation concealment mechanism	16b	Not applicable
Implementation	16c	Not applicable
Blinding (masking)	17a	Not applicable
	17b	Not applicable

For peer review only - http://bmjopen.bmj.com/site/about/guidelines.xhtml

Methods: Data collection, management, and analysis

Data collection methods	18a	Pages 7-8
	18b	Pages 7-8
Data management	19	Pages 7-8
Statistical methods	20a	Pages 10-11
	20b	Page 11
	20c	Page 11

Methods: Monitoring

Data monitoring	21a	Page 8
	21b	Not applicable
Harms	22	Not applicable
Auditing	23	Page 8

Ethics and dissemination

Research ethics approval	24	Pages 3 and 14
Protocol amendments	25	Page 14
Consent or assent (Pages 3, 11)	26a	Pages 3, 6, 9 and 14
	26b	Page 14
Confidentiality	27	Page 8 and 14
Declaration of interests	28	Page 15
Access to data	29	Page 14
Ancillary and post-trial care	30	Not applicable.

view only

Dissemination policy	31a	Page 14
	31b	Page 14
	31c	Page 14
**Appendices**		
Informed consent materials	32	Page 14
Biological specimens	33	Pages 8 and 14

For peer review only
