# Supplementary material for: Prediction of disease severity in young children presenting with acute febrile illness in resource-limited settings: a protocol for a prospective observational study
Source: BMJ Open. 2021 Jan 25;11(1):e045826. doi: 10.1136/bmjopen-2020-045826 (PMC7839891; doi:10.1136/bmjopen-2020-045826)
Supplement: Reviewer comments [file bmjopen-2020-045826.reviewer_comments.pdf]

# PEER REVIEW HISTORY

BMJ Open publishes all reviews undertaken for accepted manuscripts. Reviewers are asked to complete a checklist review form (**http://bmjopen.bmj.com/site/about/resources/checklist.pdf**) and are provided with free text boxes to elaborate on their assessment. These free text comments are reproduced below.

## ARTICLE DETAILS

| | |
|---|---|
| **TITLE (PROVISIONAL)** | Prediction of disease severity in young children presenting with acute febrile illness in resource-limited settings: a protocol for a prospective observational study |
| **AUTHORS** | Chandna, Arjun; Aderie, Endashaw; Ahmad, Riris; Arguni, Eggi; Ashley, Elizabeth; Cope, Tanya; Dat, Vu Quoc; Day, Nicholas; Dondorp, Arjen; Illanes, Victor; De Jesus, Joanne; Jimenez, Carolina; Kain, Kevin; Suy, Keang; Koshiaris, Constantinos; Lasry, Estrella; Mayxay, Mayfong; Mondal, Dinesh; Perera, Rafael; Pongvongsa, Tiengkham; Rattanavong, Sayaphet; Rekart, Michael; Richard-Greenblatt, Melissa; Shomik, Mohammad; Souvannasing, Phouthalavanh; Tallo, Veronica; Turner, Claudia; Turner, Paul; Waithira, Naomi; Watson, James A; Yosia, Mikhael; Burza, Sakib; Lubell, Yoel |

This article was not externally reviewed at BMJ Open. Protocols that have been independently assessed prior to submission to BMJ Open are usually fast-tracked to publication on the grounds that further substantial changes will not be possible. This independent assessment will usually be external, independent review for both a substantial grant award from a non-commercial or government funder and ethics approval.
